# Evaluation of microstructurally motivated constitutive models to describe age-dependent tendon healing

**DOI:** 10.1007/s10237-017-0993-4

**Published:** 2017-12-12

**Authors:** Akinjide R. Akintunde, Kristin S. Miller

**Affiliations:** Department of Biomedical Engineering, Lindy Boggs Center, Suite 500, Tulane University, New Orleans, LA 70118 USA

**Keywords:** Tendon, Aging, Healing, Constitutive model, Overparameterization, Sensitivity analysis

## Abstract

Tendon injuries are common to all ages. Injured tendons typically do not recover full functionality. The amount and organization of tendon constituents dictate their mechanical properties. The impact of changes in these constituents during (patho)physiologic processes (e.g., aging and healing) are not fully understood. Toward this end, microstructurally motivated strain energy functions (SEFs) offer insight into underlying mechanisms of age-dependent healing. Several SEFs have been adapted for tendon; however, most are phenomenological. Therefore, the aims of this study are: (1) evaluate the descriptive capability of SEFs in age-dependent murine patellar tendon healing and (2) identify a SEF for implementation in a growth and remodeling (G&R) model. To accomplish these aims, models were fitted to patellar tendon tensile data from multiple age groups and post-injury timepoints. Model sensitivity to parameters and the determinability of the parameters were assessed. A two-way analysis of variance was used to identify changes in parameters and the feasibility of implementing each model into a G&R model is discussed. The evaluated SEFs exhibited adequate descriptive capability. Parameter determinability and sensitivity analysis, however, highlighted the need for additional data to inform and validate the models to increase physiologic relevance and enable G&R model formulation to determine underlying mechanisms of age-dependent healing. This work, as a first, evaluated changes in tendon mechanical properties both as functions of age and injury in an age-dependent manner using microstructurally motivated models, highlights inherent dependencies between parameters of widely used hyperelastic models, and identified unique post-injury behavior by the aging group compared to the mature and aged groups.

## Introduction

Tendons are connective tissues that transmit muscle-generated force to bones to permit joint mobility. Further, tendons contribute to overall joint stability and protect muscles by absorbing external impact. Tendon injuries are common, debilitating, and painful disorders characterized by altered composition and structure. Available treatment strategies often fail to restore tendons to pre-injury functional capacity (Andarawis-Puri et al. 2015; Docheva et al. 2015). The underlying extracellular matrix (ECM) dynamics that drive post-injury mechanical response and influence the healing process are not fully elucidated. Further, increasing age is considered a risk factor for tendon injury and the healing potential of tendons is thought to be impaired with age (Svensson et al. 2016). The effect of aging and age-dependent healing on tendon ECM dynamics, however, is not fully elucidated.

Toward this end, the capability of growth and remodeling (G&R) models to predict (patho)physiologic processes by delineating key mechanisms of matrix dynamics is well documented (Sáez et al. 2014b; Valentín et al. 2011; Virag et al. 2015). Such models afford time- and cost-efficient frameworks to test hypotheses regarding clinical interventions such as tissue-engineering strategies (Khosravi et al. 2015; Miller et al. 2014; Niklason et al. 2010), mechanical loading regimes (Valentín et al. 2009; Valentín and Humphrey 2009b), and the critical timing of these interventions to improve clinical outcomes (Ramachandra et al. 2015). A G&R model for tendon aging and age-dependent healing requires a strain energy function (SEF) with microstructural significance in order to elucidate the salient mechanisms of age-dependent healing such as ECM production, repair, removal, damage, and organization (Humphrey and Rajagopal 2002; Lanir 2015; Rao et al. 2003; Thompson 2013). Microstructurally motivated SEFs have the capability to capture the salient response of tendons under normal and pathologic conditions, thereby aiding the understanding of underlying mechanisms through which tendons attempt to establish, maintain, or restore homeostasis.

Several SEFs have been adapted for tendons and ligaments; however, most are phenomenological (Holzapfel et al. 2000; Humphrey and Yin 1987; Weiss et al. 1996) except for the structural Shearer (SHR) model (Shearer 2015) and microstructurally motivated Gasser–Ogden–Holzapfel (GOH) model (Bajuri et al. 2016; Gasser et al. 2006). The ability of these models to describe the patellar tendon, however, have not been examined. Since significant differences in collagen organization (Thorpe et al. 2013a), function (Thorpe et al. 2012), and age-dependent and post-injury behaviors are reported between different tendons (Patel et al. 2016; Shearer et al. 2017; Thorpe et al. 2013b), it is imperative to evaluate these models’ descriptive capability in a tendon- and injury-specific manner. Additionally, the ability of the models to describe changes in tendon microstructure, organization, and mechanical properties with increasing age or in age-dependent healing have not yet been examined.

Therefore, the aims of this study are thus: (1) evaluate the descriptive capability of microstructurally motivated strain energy functions in age-dependent healing of the murine patellar tendon, and (2) based on the results of aim (1), identify (best) candidate SEF for implementation in a G&R model of murine patellar tendon healing. To identify the best candidate model, potential trade-offs or compromises that may need to be made between (a) desired level of microstructural information for a G&R model and (b) the model-to-data fit with determinable parameters (Yin et al. 1986) were evaluated. Considering the relatively large number of parameters in the SHR and GOH models, respectively, and the gross lack of data to motivate physiologic bounds for the patellar tendon with increasing age, the microstructurally motivated “Freed–Rajagopal (FR)” model (Freed and Rajagopal 2016) was also evaluated. Hence, the three models were fitted to murine patellar tendon tensile data from multiple age groups and at multiple post-injury timepoints per age group (Dunkman et al. 2013, 2014a, b; Mienaltowski et al. 2016). The sensitivity of each model to its parameters as a function of age and injury was determined through a local sensitivity analysis method. Also, within the local sensitivity method and with the correlation matrix, the determinability of the model parameters was assessed (Jaqaman and Danuser 2006). The effects of age, injury, and age–injury interaction were delineated by performing a two-way analysis of variance (ANOVA) test, followed by a post hoc test when a significant age–injury interaction was found, and the feasibility of implementing each model in a G&R model is discussed.

## Materials and methods

### Experimental data

Uniaxial load–displacement and cross-sectional area data were obtained (protocols approved by the University of Pennsylvania Institutional Animal Care and Use Committee) from published studies (Dunkman et al. 2013, 2014a, b; Mienaltowski et al. 2016) that utilized an established murine patellar tendon injury model (Beason et al. 2012; Lin et al. 2006). While details can be found in the respective publications, briefly, pre- and post-injury (at 3 and 6 weeks) quasi-static (0.1%/s) ramp-to-failure test was performed on excised murine patella-patellar tendon-tibia units. The excised units were taken from mature (120-day-old), aging (270-day-old), and aged (540-day-old) female mice. Force data were converted to first Piola–Kirchhoff stress by dividing by the undeformed cross-sectional area. Cauchy stress ($\sigma _{ zz}$) was then computed by multiplying the first Piola–Kirchhoff stress with the deformation gradient tensor component (i.e., axial stretch, $\lambda$), which was obtained from displacement data measured through optical tracking (Humphrey 2002; Taber 2004). Guided by estimated normal physiologic range of 1–4% elongation in tendons, with acute stress leading to up to ~ 8% elongation (Joìzsa 1997; Kannus 2000), a 10% elongation cutoff was applied to the stress–stretch data.

### The Shearer (SHR) SEF

Structurally, tendons are regarded as fiber-reinforced composites with collagen fibers embedded within an isotropic, non-collagenous ground matrix that consists of mainly elastin, proteoglycans, and glycosaminoglycans. In the SHR model (Shearer 2015), the ground matrix is modeled with a neo-Hookean SEF and the anisotropic mechanical behavior of the collagen fibers is captured at the fibrillar and fascicular levels. The piecewise SHR SEF is:

1 $$\begin{aligned} W^{SHR}=\left\{ {\begin{array}{ll} \left( 1-\phi \right) \frac{\mu }{2}\left( I_{1}-3 \right) &{}\quad I_{4}<1\, \\ \left( 1-\phi \right) \frac{\mu }{2}\left( I_{1}-3 \right) + &{} \\ \phi \frac{E}{3\sin ^{2}\theta _{0}}\left( 2\cos {\alpha \sqrt{I_{4}} }-3\ln \left( 2\left( \cos ^{2}{\alpha \sqrt{I_{4}} }\right. \right. \right. &{}\\ \quad \left. \left. \left. +\cos {\alpha \sqrt{\Gamma }} \right) \right) +\frac{\cos {\alpha \sqrt{I_{4}}}}{\sin ^{2}{\alpha \sqrt{\Gamma } }} \right) +\gamma &{}\quad 1\le I_{4}\le \lambda ^{*^{2}} \\ \left( 1-\phi \right) \frac{\mu }{2}\left( I_{1}-3 \right) +\phi E\left( \beta \cos {\alpha \sqrt{I_{4}} } \right) &{} \\ \quad -\ln \left( \cos ^{2}{\alpha \sqrt{I_{4}}}+\cos {\alpha \sqrt{{\Gamma }} } \right) +\eta &{}\quad I_{4}>\lambda ^{*^{2}} \\ \end{array}} \right. \quad \end{aligned}$$

where $\phi \, \epsilon \, (0, 1]$ is the collagen fiber volume fraction, $\mu$ is the shear modulus of the ground matrix. $I_{1}=tr({\mathbf {C}})$ and $I_{4}=\vec {M}{\mathbf {C}}\vec {M}$ are the first and fourth invariants of the right Cauchy–Green tensor $({\mathbf {C}}={\mathbf {F}}^{\mathrm{T}}{\mathbf {F}})$, respectively, where $\vec {M}$ is the preferred direction in the reference configuration and ${\mathbf {F}}$ is the deformation gradient tensor. *E* is the fibril Young’s modulus, $\theta _{0}$ is the crimp angle of the outermost fibrils, $\alpha$ is the fibril helix angle, and ${\Gamma =}\sin ^{2}\alpha +I_{4}\cos ^{2}\alpha$. The stretch in the fascicle direction that straightens outer fibrils (transition stretch), $\lambda ^{*}$, and the parameters $\beta$, $\gamma$ and $\eta$ are defined as follows:

2 $$\begin{aligned}&\lambda ^{*}=\frac{1}{\cos \alpha }\sqrt{\frac{1}{\cos ^{2}\theta _{o}}-\sin ^{2}\alpha } \end{aligned}$$

3 $$\begin{aligned}&\beta =\frac{2\left( 1-\cos ^{3}\theta _{o} \right) }{3\sin ^{2}\theta _{o}} \end{aligned}$$

4 $$\begin{aligned}&\gamma =-\frac{E}{3\sin ^{2}\theta _{o}}\left[ 2\cos \alpha -3\ln \left( \cos ^{2}\alpha +\cos \alpha \right) +\frac{\cos \alpha }{\sin ^{2}\alpha } \right] \nonumber \\\end{aligned}$$

5 $$\begin{aligned}&\eta =\gamma +E\left[ \left( \frac{\cos \theta _{o}}{\sin ^{2}\theta _{o}}\frac{1}{\sin ^{2}\alpha }+\frac{2}{\sin ^{2}\theta _{o}}-3\beta \right) \lambda ^{*}\cos \alpha \right. \nonumber \\&\quad \left. \quad -3\tan ^{2}\theta _{o}\ln \left( \frac{\cos \alpha }{\cos \theta _{o}}+\lambda ^{*}\cos ^{2}\alpha \right) \right] \end{aligned}$$

### The Gasser–Ogden–Holzapfel (GOH) SEF

The patellar tendon, for simplicity, was assumed to consist of a single family of collagen fibers with a preferred direction represented by $\vec {M}^\mathrm{GOH}$ (which makes an angle, $\xi$, with the long axis of the tendon and can be determined through histology) with axial and radial components in the undeformed configuration. The GOH model uniquely captures the dispersion of fibers around $\vec {M}^\mathrm{GOH}$ within the fiber family via the experimentally measurable structural parameter, $\kappa \, \epsilon \, [0,\, 1/3]$ (Gasser et al. 2006). When fibers are perfectly aligned along $\vec {M}^\mathrm{GOH}$, $\kappa = 0$ and when the fibers are distributed isotropically, $\kappa = 1/3$. The non-collagenous ground matrix is represented by the neo-Hookean SEF. The SEF for the whole tendon is represented by

6 $$\begin{aligned} W^\mathrm{GOH}= & {} \left( 1-\phi \right) \frac{\mu }{2}\left( I_{1}-3 \right) \nonumber \\&+\,\phi \frac{c_{1}^\mathrm{c}}{c_{2}^\mathrm{c}}\left[ \hbox {exp}\left\{ c_{2}^\mathrm{c}\Big [ \kappa I_{1}+\left( 1-3\kappa \right) I_{4}-1 \right] ^{2} \right\} -1 \Bigg ]\nonumber \\ \end{aligned}$$

$\phi$, $\mu$, $I_{1}$ and $I_{4}$ have been defined previously (see Sect. 2.2), $c_{1}^\mathrm{c}>0$ is a modulus-like parameter, and $c_{2}^\mathrm{c}>0$ is a dimensionless parameter. The GOH model contains collagen-related parameters ($c_{1}^\mathrm{c}$ and $c_{2}^\mathrm{c}$) that capture collagen fiber behavior phenomenologically, but whose physical significance is unclear and subject to personal interpretation as neither parameter can be measured experimentally. Such inherent limitation in phenomenological models motivated the development of the SHR model.

### Constitutive formulation for the SHR and GOH models

For simplicity, the patellar tendon was assumed to have a solid cylindrical geometry and to be incompressible—a valid assumption for tendon in quasi-static extension (Dourte et al. 2013; Vergari et al. 2011). Hence, the deformation gradient tensor and the resulting right Cauchy–Green tensor are:

7 $$\begin{aligned}&{\mathbf {F}}=\left[ {\begin{array}{*{20}c} \lambda ^{-1 / 2} &{} 0 &{} 0\\ 0 &{} \lambda ^{-1 / 2} &{} 0\\ 0 &{} 0 &{} \lambda \\ \end{array} } \right] \Longrightarrow {\mathbf {C}}=\left[ {\begin{array}{*{20}c} \lambda ^{-1} &{} 0 &{} 0\\ 0 &{} \lambda ^{-1} &{} 0\\ 0 &{} 0 &{} \lambda ^{2}\\ \end{array} }\right] \end{aligned}$$

8 $$\begin{aligned}&\vec {M}^\mathrm{SHR}=\left[ \sin \, \psi ,\, 0,\, \cos \psi \right] , \quad \vec {M}^\mathrm{GOH}=\left[ \sin \, \xi ,\, 0,\, \cos \xi \right] \nonumber \\ \end{aligned}$$

where $\lambda$ is the stretch measured along the long axis of the tendon during the uniaxial extension test. In cylindrical coordinates, $\vec {M}^\mathrm{SHR}$ is the preferred fascicle direction, and $\psi$ is similar to GOH’s $\xi$, but at the fascicular level. The strain invariants (defined in Sect. 2.2) can then be expressed as: $I_{1}=2\left( \lambda ^{-1} \right) +\lambda ^{2}$, $I_{4}^\mathrm{SHR}=\lambda ^{-1}\sin ^{2}\psi +\lambda ^{2}\cos ^{2}\psi ,\,$ and $I_{4}^\mathrm{GOH}=\lambda ^{-1}\sin ^{2}\xi +\lambda ^{2}\cos ^{2}\xi$. The constitutive framework for transverse isotropy (Ogden 2003) was used, where $W=\hat{W}\left( I_{1},I_{4} \right)$ as is the case with the SHR and GOH models,

9 $$\begin{aligned} {{\varvec{\sigma }}}=-p{\mathbf {I}}+2\frac{\partial W}{\partial I_{1}}{\mathbf {b}}+2\frac{\partial W}{\partial I_{4}}{\mathbf {F}}\, \vec {M}\, \otimes \, {\mathbf {F}}\, \vec {M} \end{aligned}$$

where ${\mathbf {b}=\mathbf {F}}{\mathbf {F}}^{\mathrm{T}}$ is the left Cauchy–Green tensor, numerically equal to ${\mathbf {C}}$. Within this framework, for the SHR model, one obtains the following:

10 $$\begin{aligned} \sigma _{ zz}^\mathrm{SHR}= & {} \left( 1-\phi \right) \mu \left( \lambda ^{2}-\frac{1}{\lambda } \right) \nonumber \\&+\,2\chi ^\mathrm{SHR}\left( \lambda ^{2}\cos ^{2}\psi -\frac{\sin ^{2}\psi }{2\lambda } \right) \end{aligned}$$

where,

11 $$\begin{aligned} \chi ^\mathrm{SHR}=\left\{ {\begin{array}{ll} 0 &{}\quad I_{4}<1 \\ \phi \frac{E\cos \alpha }{6\sqrt{I_{4}} \sin ^{2}\theta _{0}}\left[ 2-\left( \frac{3{\Gamma }-1}{{\Gamma }\sqrt{\Gamma } } \right) \right] &{}\quad 1\le I_{4}\le \lambda ^{*^{2}} \\ \phi \frac{E\cos \alpha }{2\sqrt{I_{4}}}\left( \beta -\frac{1}{\sqrt{{\Gamma }} } \right) &{}\quad I_{4}>\lambda ^{*^{2}} \\ \end{array}} \right. \end{aligned}$$

Preliminary computational studies on the experimental data showed that the fibril helix angle, $\alpha \approx 0$. This was also alluded to by Shearer for the human patellar tendon (Shearer 2015). Hence, the same was assumed valid for the murine patellar tendon, and $\chi ^\mathrm{SHR}$ then reduces to:

12 $$\begin{aligned} \chi ^\mathrm{SHR}=\left\{ {\begin{array}{ll} 0 &{}\quad I_{4}<1 \\ \phi \frac{E}{6\sqrt{I_{4}} \sin ^{2}\theta _{0}}\left[ 2-\left( \frac{3I_{4}-1}{I_{4}\sqrt{I_{4}}} \right) \right] &{}\quad 1\le I_{4}\le \lambda ^{*^{2}} \\ \phi \frac{E}{2\sqrt{I_{4}}}\left( \beta -\frac{1}{\sqrt{I_{4}}} \right) &{}\quad I_{4}>\lambda ^{*^{2}}\end{array}} \right. \, \end{aligned}$$

Similarly, one obtains the following expression for the GOH model axial Cauchy stress ($\sigma _{ zz}^\mathrm{GOH}$), having found the Lagrange multiplier, *p* through equilibrium equation ($\vec {\nabla }\cdot \varvec{\sigma }=\vec {0}$) and application of no-traction boundary condition at the tendon’s outer radial surface:

13 $$\begin{aligned} \sigma _{ zz}^\mathrm{GOH}= & {} \left( 1-\phi \right) \mu \left( \lambda ^{2}-\frac{1}{\lambda } \right) +\chi ^\mathrm{GOH}\left[ \kappa \left( \lambda ^{2}-\frac{1}{\lambda } \right) \right. \nonumber \\&\left. +\left( 1-3\kappa \right) \left( \lambda ^{2}\cos ^{2}\xi -\frac{\sin ^{2}\xi }{2\lambda } \right) \right] \end{aligned}$$

where $$\chi ^\mathrm{GOH}=4\phi c_{1}^\mathrm{c}ve^{c_{2}^\mathrm{c}v^{2}}$$ and $$v=\left[ \kappa I_{1}+\left( 1-3\kappa \right) I_{4}-1 \right] $$.

### The Freed–Rajagopal (FR) constitutive model

The Freed–Rajagopal (FR) model captures the strain-limiting behavior of biological fibers. The total fiber strain is decomposed into that of collagen fibrils $(\varepsilon ^\mathrm{C})$ that behave as Hookean fibers added to a network of elastin filaments $(\varepsilon ^\mathrm{E})$ that behave as strain-limiting fibers whose stress–strain relationship is derived from an implicit SEF (Freed and Rajagopal 2016). The model has three parameters with physical significance. The parameters are: the elastic modulus at zero strain (i.e., toe region modulus ($E^\mathrm{E}>0)$), the elastic modulus of the linear region, $E^\mathrm{C}$ ($>E^\mathrm{E}$), and $\beta =1 /\varepsilon _\mathrm{max}^\mathrm{crimp}$, which is the reciprocal of the transition true strain (the value at which all collagen fibers are fully engaged). The superscripts *E* and *C* indicate “elastin-controlled” and “collagen-controlled,” respectively. The FR model equations are:

14 $$\begin{aligned} \lambda= & {} \lambda ^\mathrm{E}\lambda ^\mathrm{C}\, \, \, \quad \varepsilon =\ln \lambda \quad \, \, \, \varepsilon =\varepsilon ^\mathrm{E}+\varepsilon ^\mathrm{C} \end{aligned}$$

15 $$\begin{aligned} \varepsilon= & {} \frac{1}{\beta }\left\{ 1-\frac{1}{\left[ 1+\left( \beta -1 \right) \frac{\sigma _{ zz}^\mathrm{exp}}{E^\mathrm{E}} \right] ^{\beta /\beta -1}}\right\} +\frac{\sigma _{ zz}^\mathrm{exp}}{E^\mathrm{C}} \end{aligned}$$

### Constraining the model parameters

To ensure that physiologically relevant, optimized model parameter values are obtained; model parameters must be constrained to bounds informed by experimental data. Such experimental data, particularly structural data, are not readily available for most tendons and animal species. Moreover, the experimental datasets to which models were fitted herein cover multiple age groups and healing timepoints. Hence, the histological data that could inform appropriate bounds for the model parameters (e.g., $\kappa$—collagen dispersion parameter and $\theta _{0}$—collagen fibril crimp angle) at each age and healing timepoint are not readily available to the authors’ knowledge. Consequently, reasonable theoretical bounds were placed on all the structural parameters (Table 1).

**Table 1:** Theoretically and experimentally motivated constraints placed on model parameters during the data fitting process

Model	Parameter	Constraint	References
*Material*			
GOH & SHR	$({1-\phi })\mu$	$>\,0$	
GOH	$$\phi c_1^c $$ and $$c_2^c $$	$$>\,0$$	
SHR	$\phi E$	0–14 GPa	Andriotis et al. (2015)
FR	$E^\mathrm{C}$	$>\,0$	
FR	$E^\mathrm{E}$	$>\,0$	
*Structural*			
GOH	$\kappa$	0–1/3	Gasser et al. (2006)
GOH	$\xi$	0–$\pi /2$	
SHR	$\theta _0$	0–$\pi /2$	Shearer (2015)
SHR	$\psi$	0–$\pi /2$	
FR	$\beta$	10–1000	

Experimental elongation values range from 1 to 10% (see Sect. 2.1); hence, $\beta$ was bounded by values corresponding to the limits of this range. For both SHR and GOH models, the ground substance shear modulus was required to be positive. GOH model collagen-related material parameters ($\phi c_{1}^\mathrm{c}$ and $c_{2}^\mathrm{c}$), and the FR model moduli ($E^\mathrm{C}$ and $E^\mathrm{E}$) were required to be positive. A wide range of collagen fibril moduli have been reported from atomistic computational models (Gautieri et al. 2011), as well as experimental measurements on fibrils from sea cucumber (Eppell et al. 2006), rats (Dutov et al. 2016; Wenger et al. 2007), and cows (Van Der Rijt et al. 2006). Herein, upper bound for the SHR model’s $\phi E$ (Young’s modulus of collagen fibrils) was set at 14 GPa (Andriotis et al. 2015). This value is the mean plus two times the standard deviation for indentation modulus (with contributions from longitudinal and transverse moduli) measured in collagen fibrils of skeletally mature mice (Brodt et al. 1999). Bounds for all model parameters are listed in Table 1.

### Data fitting

The models (Eqs. 10, 13, 15) are nonlinear in the model parameters leading to nonlinear optimization problems. The function *lsqnonlin* in MATLAB (The MathWorks, Inc, Natick, MA, USA) was employed with the trust-region-reflective algorithm. It minimizes an objective function $f\left( \vec {x} \right)$ in a least squares sense $\left( \sum \nolimits _n \left[ f\left( \vec {x} \right) \right] ^{2} \right)$. The optimal objective function chosen provides a good compromise between low and high strains (Ferruzzi et al. 2013):

16 $$\begin{aligned} f^{\mathrm{GOH/SHR}}\left( \vec {x} \right) =\frac{\sigma _{ zz}^\mathrm{th}\left( \vec {x} \right) -\sigma _{ zz}^\mathrm{exp}}{\bar{\sigma }_{ zz}^\mathrm{exp}} \end{aligned}$$

where $\vec {x}$ is a vector of the model parameters with each component subjected to lower and upper bounds. $\sigma _{ zz}^\mathrm{th}\left( \vec {x} \right)$ and $\sigma _{ zz}^\mathrm{exp}$ are the theoretical (th) and experimental (exp) axial Cauchy stresses, respectively, and $\bar{\sigma }_{ zz}^\mathrm{exp}$ is the average experimental axial Cauchy stress. *n* is the number of data points in the experimental data pair per specimen. Similarly, for the FR model,

17 $$\begin{aligned} f^\mathrm{FR}(\vec {x})=\frac{\varepsilon _{ zz}^\mathrm{th}( \vec {x} )-\varepsilon _{ zz}^\mathrm{exp}}{\bar{\varepsilon }_{ zz}^\mathrm{exp}} \end{aligned}$$

where $\varepsilon _{ zz}^\mathrm{th}\left( \vec {x} \right)$ and $\varepsilon _{ zz}^\mathrm{exp}$ are the theoretical (th) and experimental (exp) axial true strains, respectively, and $\bar{\varepsilon }_{ zz}^\mathrm{exp}$ is the average experimental axial true strain.

**Fig. 1:**
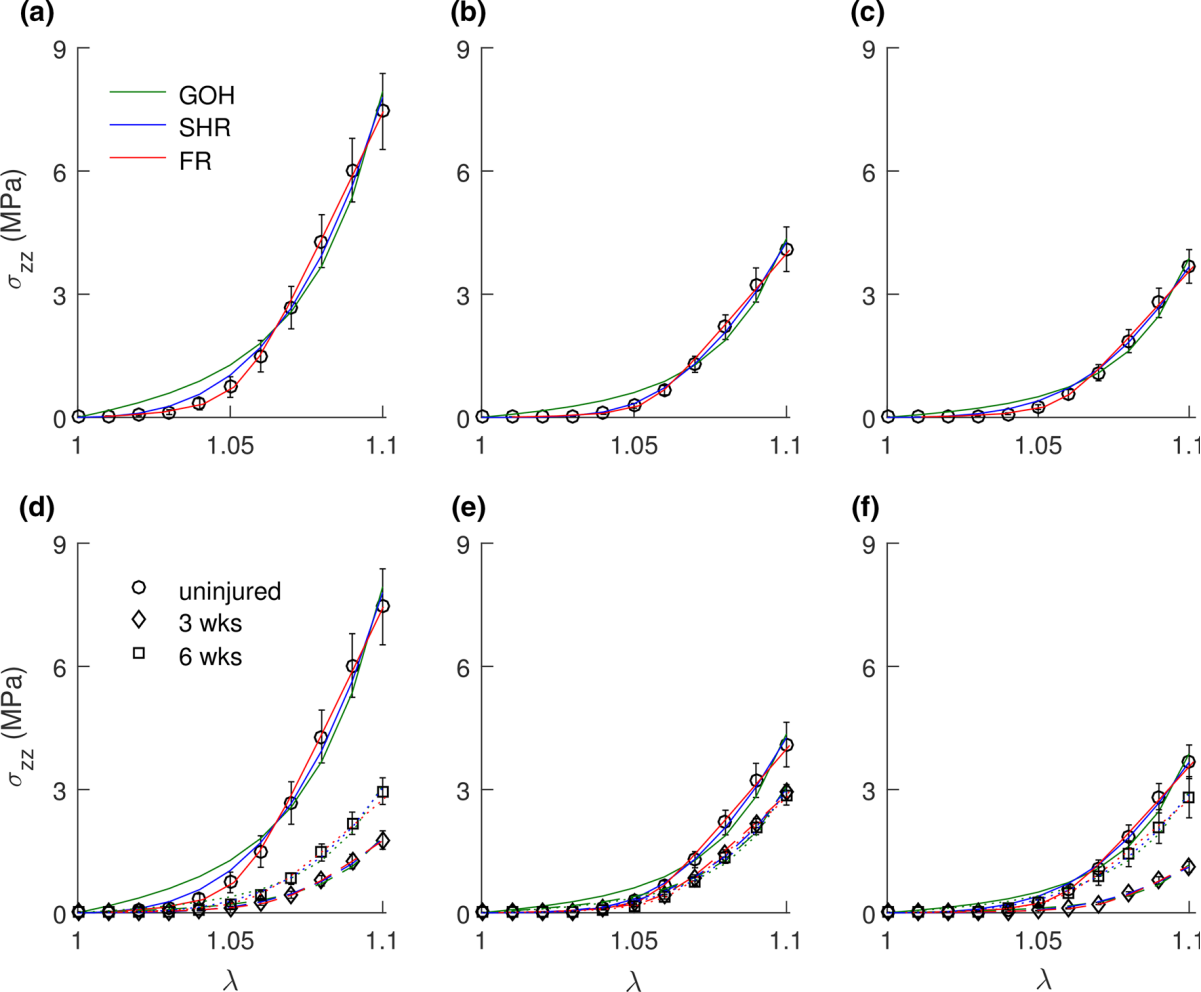
Fits of the SHR (blue), GOH (green), and FR (red) models to experimental tensile test data [mean (open symbols) ± SE (bar)] for the murine patellar tendons. Top row (aging): **a** mature, **b** aging, and **c** aged. Bottom row (age-dependent healing): **d** mature, **e** aging, and **f** aged. $\hbox {Solid\,line}= \hbox {uninjured}$, $\hbox {dashed\,line}= 3\,\hbox {week}$, and $\hbox {dotted\,line} = \hbox {6\,weeks}$. Models exhibited reasonable fits to the experimental data. A decline with age in the ultimate tensile strength of the tendons is observable from the top row

To ensure parameter independence from initial guess values (i.e., global not local minima), random numbers were set as initial guesses for three fits over each specimen data using the *MultiStart* MATLAB function, and the average of model parameter values resulting from the multiple fits of each experimental dataset was taken as best-fit value for each model parameter.

### Model sensitivity analysis and parameter determinability

The rationale for a sensitivity analysis on the three models is: (1) To determine which parameters are most influential to the uniaxial mechanical response of uninjured tendons and (2) to elucidate alterations in parameter influence across age groups and post-injury. To accomplish this, the local method for sensitivity analysis (Hamby 1994) was used by computing the dimensionless sensitivity coefficients (stretch-dependent derivatives), $s\left( \lambda \right)$. This local method has been used in the assessment of constitutive models for rubber (Ogden et al. 2004) and soft tissues (Fink et al. 2008; Weisbecker et al. 2015). The dimensionless sensitivity coefficients for the GOH and SHR models were computed as:

18 $$\begin{aligned} s_{i}^{\mathrm{GOH/SHR}}\left( \lambda \right) =\frac{\partial \sigma _{ zz}^{\mathrm{GOH/SHR}}\left( \lambda \right) }{\partial x_{i}}\frac{x_{i}}{\sigma _{ zz}^{\mathrm{GOH/SHR}}\left( \lambda \right) } \end{aligned}$$

and for the FR model as:

19 $$\begin{aligned} s_{i}^\mathrm{FR}\left( \lambda \right) =\frac{\partial \varepsilon ^\mathrm{FR}\left( \lambda \right) }{\partial x_{i}}\frac{x_{i}}{\varepsilon ^\mathrm{FR}\left( \lambda \right) } \end{aligned}$$

where $x_{i}~( i=1,2,3,\ldots )$ are the model parameters. In this method, two parameters are considered dependent if their sensitivity plots are similar in shape, which suggests that the parameters cannot be uniquely determined through the optimization process. Hence, one parameter should be determined prior to model initiation (e.g., fixed based on experimental data outside that which was fitted to the model). To further ascertain dependency (i.e., parameter determinability) among the model parameters, the correlation matrix was computed (Yin et al. 1986). It contains the correlation coefficients between parameters and has values ranging from − 1 to $+\,1$, where 0 indicates no dependency and $\pm \, 1$ indicates total dependency (Jaqaman and Danuser 2006). Model sensitivity analysis was performed using model parameters for the average experimental data for each age group and pre- and post-injury subgroups.

### Statistical analysis

A two-way ANOVA was used to simultaneously determine the effects of age (mature, aging, aged), injury (uninjured, 3 week post-injury, 6 week post-injury), and their interaction on each model parameter. The number of observations per cell (constituted by each age–injury combination) was not the same (*n* ranged from 5 to 20 per timepoint within each age group), yielding an unbalanced factorial design. Type I sum of squares weighs all cells equally, thus is not appropriate in this situation. This leaves the choice between types II and III (the correction methods for imbalance). The choice of type II is advocated when no interaction effect between the factors is possible, which is not the case herein (King 2016; Landsheer and van den Wittenboer 2016). Hence, a type III sum of squares was used. Note that the use of type III is advised when there is possibility of interaction, as it has been shown to generate modest estimates with infinitesimally small probability of main effect overestimation, unlike type II (Landsheer and van den Wittenboer 2016). Statistical significance was set at $p\le 0.05$, and statistical trend at $0.05 < p \le 0.1$. The Holm–Sidak post hoc test was performed on parameters that exhibited significance in the age–injury interaction. The effects of age were studied by performing the post hoc test on a group composed of all the uninjured samples across all age groups. Also, the post hoc test was performed on all the mature, aging, and aged groups to investigate the age-dependent effects on healing within each age group. All statistical analyses were performed in Team RC (2016).

## Results

### Data fitting

**Fig. 2:**
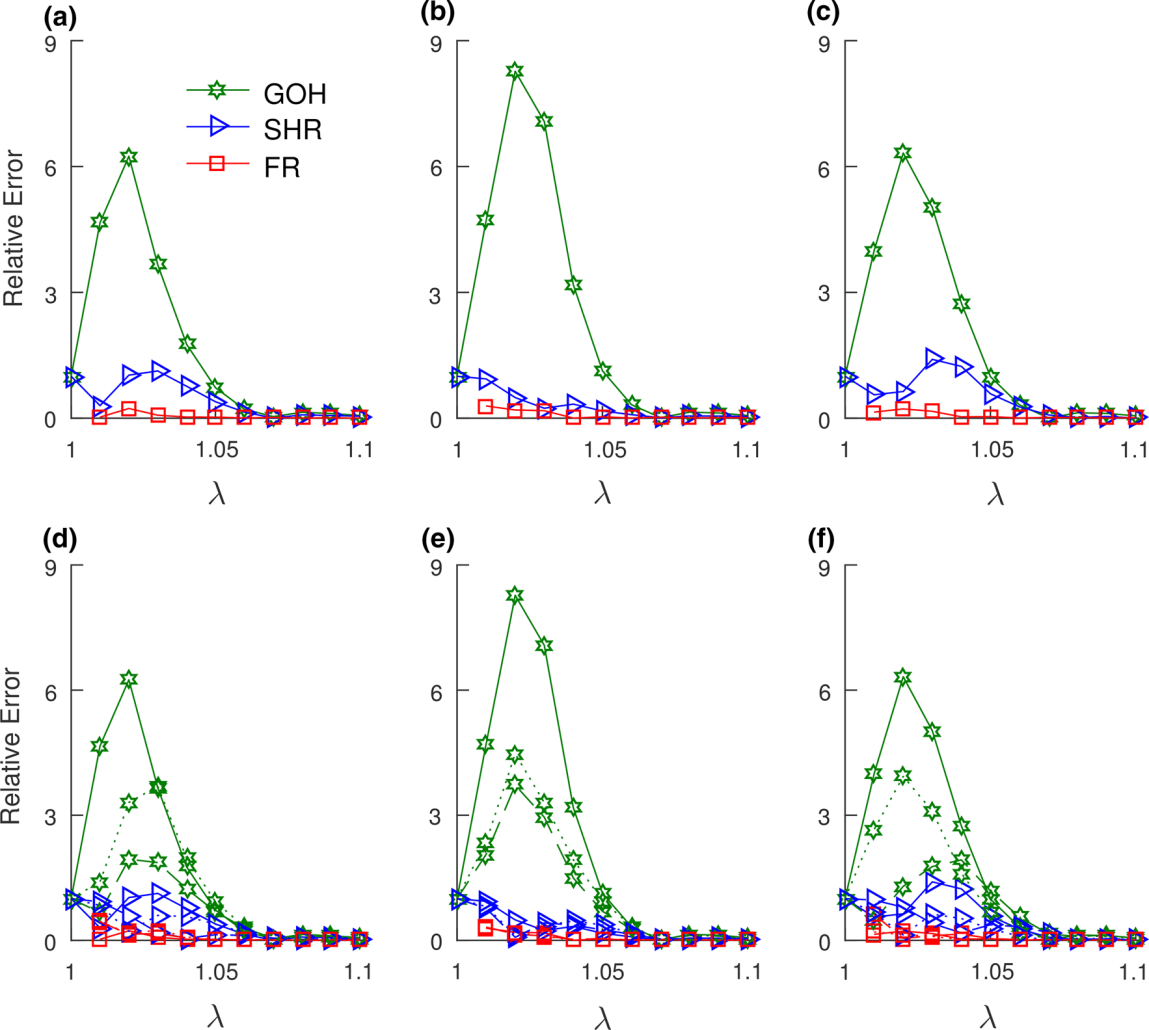
Relative errors of SHR (blue), GOH (green), and FR (red) models. Top row: **a** mature, **b** aging, and **c** aged, all uninjured. Bottom row: **d** mature, **e** aging, and **f** aged—solid lines represents uninjured, dashed lines represents 3 weeks and dotted lines represents 6 weeks. For all models, larger relative errors occurred in the toe region. The FR model had the least relative error values

The goodness-of-fit (GOF) values, $R^{2}$ (presented as mean ± standard error (SE)), for the model are SHR: $0.992 \pm 0.001$, GOH: $0.976 \pm 0.001$, and FR: $0.942 \pm 0.008$. All three models exhibited acceptable fits to the experimental data for all age groups and post-injury timepoints (Fig. 1). The average optimized model parameters for all age groups and healing timepoints are listed for the SHR, GOH, and FR models in Tables 2, 4, and 6, respectively. The relative errors for the three models were plotted as functions of axial stretch to assess the accuracy of the models over the toe, transition, and linear regions of the stress–stretch curve (Fig. 2). As is commonly observed for hyperelastic materials (Ogden et al. 2004), the relative errors were largest in the toe region (Fig. 2). The relative errors associated with the GOH model were the largest, while the FR model exhibited the smallest relative errors. As a test of convergence of the three runs of the local solver through the *Multistart* function the coefficient of variation (CV) of the three FR parameters where computed for the uninjured mature group (${n} = 5$). CV within the group for the three runs had a range of 0.03–5.43%.

**Table 2:** SHR model parameters (mean ± SE) obtained from the data fitting process with $\psi [{0^{\circ },90^{\circ }}]$

Group	$(1-\phi )\mu ~({\mathrm{kPa}})$	$\phi E~( {\mathrm{GPa}})$	$^{(\# \mathrm{ai})} \theta _o~(^{\circ })$	$^{(*{\mathrm{a}}, *\mathrm{i}, *\mathrm{ai})}\psi ~(^{\mathrm{o}})$	$R^{2}$
*Mature*					
Ctl	$0.98\pm 0.37$	$8.43\pm 0.34$	$14.6\pm 4.6$	$50.2\pm 1.6$	$0.986\pm 0.002$
3 weeks	$0.48\pm 0.13$	$7.27\pm 0.98$	$11.9\pm 1.9$	$53.0\pm 0.5$	$0.988\pm 0.006$
6 weeks	$0.71\pm 0.14$	$7.32\pm 0.96$	$10.0\pm 1.5$	$52.9\pm 0.5$	$0.994\pm 0.001$
*Aging*					
Ctl	$0.79\pm 0.16$	$9.97\pm 0.77$	$10.8\pm 1.4$	$52.7\pm 0.4$	$0.989\pm 0.002$
3 weeks	$0.64\pm 0.12$	$9.10\pm 1.03$	$13.3\pm 1.8$	$52.2\pm 0.5$	$0.993\pm 0.001$
6 weeks	$1.51\pm 0.61$	$7.31\pm 0.83$	$8.8 \pm 1.0$	$53.3\pm 0.3$	$0.994\pm 0.001$
*Aged*					
Ctl	$0.85\pm 0.14$	$8.85\pm 0.63$	$9.8 \pm 1.5$	$53.0\pm 0.5$	$0.990\pm 0.002$
3 weeks	$4.22\pm 3.70$	$8.91\pm 1.23$	$8.4 \pm 1.2$	$54.5\pm 0.2$	$0.995\pm 0.001$
6 weeks	$0.86\pm 0.16$	$8.86\pm 1.22$	$13.8\pm 2.5$	$52.6\pm 0.7$	$0.995\pm 0.001$

$^{*\mathrm{a}}$Significance with age ($p <0.05$); $^{*\mathrm{i}}$significance with injury ($p <0.05$); $^{*\mathrm{ai}}$significant age–injury interaction ($p<0.05$); $^{\#\mathrm{ai}}$trend in age–injury interaction ($p<0.1$)

**Table 3:** SHR model parameters (mean ± SE) obtained from the data fitting process with $\psi \left[ {0^{\circ },10^{\circ }} \right]$

Group	$(1-\phi )\mu ~({\mathrm{kPa}})$	$^{(*\mathrm{a}, *\mathrm{i}, *\mathrm{ai})}\phi E~({\mathrm{MPa}})$	$^{(*\mathrm{ai})}\theta _o~(^{{\circ }})$	$^{(*\mathrm{ai})}\psi ~(^{\mathrm{o}})$	$R^{2}$
*Mature*					
Ctl	$0.01\pm 0.001$	$217.74\pm 32.33$	$27.1\pm 2.6$	$7.5\pm 0.6$	$0.892\pm 0.028$
3 weeks	$0.09\pm 0.04$	$11.12 \pm 5.26$	$10.6\pm 3.9$	$3.3\pm 1.0$	$0.842\pm 0.014$
6 weeks	$1.13\pm 0.76$	$52.88 \pm 16.08$	$12.9\pm 3.9$	$3.4\pm 0.8$	$0.855\pm 0.015$
*Aging*					
Ctl	$0.13\pm 0.06$	$32.71\pm 12.81$	$10.7\pm 3.5$	$2.9\pm 0.6$	$0.852\pm 0.018$
3 weeks	$0.05\pm 0.02$	$67.15\pm 9.74$	$25.5\pm 3.0$	$5.5\pm 0.6$	$0.868\pm 0.015$
6 weeks	$0.03\pm 0.01$	$41.75\pm 11.12$	$16.8\pm 4.1$	$4.2\pm 0.7$	$0.850\pm 0.011$
*Aged*					
Ctl	$0.08\pm 0.06$	$63.05\pm 16.43$	$16.7\pm 3.5$	$3.7\pm 0.6$	$0.834\pm 0.015$
3 weeks	$0.14\pm 0.05$	$12.46\pm 6.38$	$7.6 \pm 3.7$	$3.3\pm 1.2$	$0.800\pm 0.022$
6 weeks	$0.24\pm 0.22$	$54.75\pm 21.34$	$10.7\pm 3.7$	$3.4\pm 1.0$	$0.862\pm 0.019$

$^{*\mathrm{a}}$Significance with age ($p <0.05$); $^{*\mathrm{i}}$significance with injury ($p <0.05$); $^{*\mathrm{ai}}$significant age–injury interaction ($p<0.05$)

The relatively large optimized values taken by the fascicle alignment, $\psi$ (Table 2) and the preferred collagen fiber, $\xi$ (Table 4) parameters for the SHR and GOH models, respectively, were surprising, considering that the patellar tendon has longitudinally aligned fibers and measurements in the porcine patellar tendon have shown these values to be less than ${10}^{\circ }$ (Shearer et al. 2014). Hence, parameter optimization was repeated for the SHR and GOH models in all experimental groups, with the parameters—$\psi$ and $\xi$ bounded within the range $0^{\circ }$–${10}^{\circ }$. Also, guided by experimental measurements in mice and rat tails, and human Achilles tendon (Diamant et al. 1972; Legerlotz et al. 2014), the fibril crimp angle, $\theta _{o}$ was set within the range $0^{\circ }$–${45}^{\circ }$. The remaining parameter bounds used were the same as in the prior study (Table 1). With these revised bounds, the GOF values decreased for the SHR model to $0.848 \pm 0.006$ and to $0.964 \pm 0.011$ for the GOH model. Optimized parameter values obtained with the revised constraints are listed for the SHR (Table 3), and GOH (Table 5) models.

**Table 4:** GOH model parameters (mean ± SE) obtained from the data fitting process with $\xi [ {0^{\circ }, 90^{\circ }}]$

Group	$$(1-\phi )\mu ({\mathrm{kPa}})$$	$$\phi c_1^c~({\mathrm{MPa}})$$	$$c_2^c $$	$$^{(*\mathrm{a},*\mathrm{i}, *\mathrm{ai})}\kappa $$	$$\xi ~(^{{\circ }})$$	$$R^{2}$$
*Mature*						
Ctl	$0.51\pm 0.19$	$0.94\pm 0.94$	$347\pm 150$	$0.146\pm 0.025$	$11.5\pm 1.5$	$0.956\pm 0.004$
3 weeks	$0.58\pm 0.22$	$0.12\pm 0.05$	$353\pm 88$	$0.039\pm 0.009$	$18.7\pm 4.0$	$0.984\pm 0.001$
6 weeks	$0.46\pm 0.09$	$0.06\pm 0.05$	$426\pm 90$	$0.054\pm 0.010$	$20.8\pm 5.2$	$0.981\pm 0.002$
*Aging*						
Ctl	$0.37\pm 0.12$	$0.13\pm 0.08$	$257\pm 70$	$0.036\pm 0.008$	$9.0 \pm 2.0$	$0.971\pm 0.004$
3 weeks	$1.20\pm 0.48$	$1.94\pm 1.55$	$237\pm 63$	$0.052\pm 0.009$	$16.3\pm 4.2$	$0.974\pm 0.001$
6 weeks	$0.39\pm 0.08$	$0.13\pm 0.10$	$522\pm 103$	$0.070\pm 0.013$	$13.8\pm 3.4$	$0.975\pm 0.002$
*Aged*						
Ctl	$1.39\pm 0.81$	$2.50\pm 1.39$	$188\pm 64$	$0.041\pm 0.008$	$24.6\pm 5.6$	$0.973\pm 0.003$
3 weeks	$0.53\pm 0.23$	$0.13\pm 0.07$	$364\pm 121$	$0.054\pm 0.012$	$17.7\pm 5.4$	$0.981\pm 0.003$
6 weeks	$0.32\pm 0.12$	$0.38\pm 0.24$	$227\pm 73$	$0.038\pm 0.006$	$13.6\pm 4.3$	$0.982\pm 0.003$

$^{*\mathrm{a}}$Significance with age ($p<0.01$); $^{*\mathrm{i}}$significance with injury ($p<0.05$); $^{*\mathrm{ai}}$significant age–injury interaction ($p<0.001$)

**Table 5:** GOH model parameters (mean ± SE) obtained from the data fitting process with $\xi [{0^{\circ }, 10^{\circ }}]$

Group	$$(1-\phi )\mu ~({\mathrm{kPa}} )$$	$$\phi c_1^c~({\mathrm{MPa}})$$	$$c_2^c $$	$$\kappa $$	$$^{(*\mathrm{i})}\xi ~(^{{\circ }})$$	$$R^{2}$$
*Mature*						
Ctl	$0.40\pm 0.09$	$0.08\pm 0.08$	$354\pm 135$	$0.058\pm 0.018$	$3.4\pm 0.6$	$0.956\pm 0.004$
3 weeks	$0.40\pm 0.10$	$0.26\pm 0.13$	$323\pm 65$	$0.073\pm 0.009$	$2.8\pm 0.5$	$0.980\pm 0.001$
6 weeks	$0.29\pm 0.15$	$0.62\pm 0.28$	$221\pm 88$	$0.070\pm 0.018$	$3.2\pm 0.5$	$0.979\pm 0.003$
*Aging*						
Ctl	$3.60\pm 2.45$	$0.77\pm 0.30$	$197\pm 85$	$0.061\pm 0.018$	$4.7\pm 0.4$	$0.969\pm 0.003$
3 weeks	$0.66\pm 0.30$	$0.46\pm 0.17$	$233\pm 69$	$0.058\pm 0.011$	$3.4\pm 0.3$	$0.974\pm 0.001$
6 weeks	$1.19\pm 0.84$	$0.50\pm 0.17$	$140\pm 69$	$0.037\pm 0.010$	$3.9\pm 0.5$	$0.975\pm 0.002$
*Aged*						
Ctl	$1.52\pm 0.78$	$0.46\pm 0.18$	$175\pm 66$	$0.045\pm 0.012$	$3.5\pm 0.4$	$0.907\pm 0.065$
3 weeks	$0.04\pm 0.03$	$0.10\pm 0.01$	$42 \pm 11$	$0.016\pm 0.008$	$2.1\pm 0.7$	$0.979\pm 0.003$
6 weeks	$0.89\pm 0.51$	$0.46\pm 0.28$	$271\pm 105$	$0.064\pm 0.013$	$4.6\pm 0.5$	$0.982\pm 0.003$

$^{*\mathrm{i}}$Significance with injury ($p<0.05$)

**Table 6:** FR model parameters (Mean ± SE) obtained from the data fitting process

Group	$^{(*\mathrm{a},*\mathrm{i},*\mathrm{ai})}E^\mathrm{E}~(\mathrm{MPa})$	$^{(*\mathrm{a},*\mathrm{i}, *\mathrm{ai})}E^\mathrm{C}~({\mathrm{MPa}})$	$\beta$	$R^{2}$
*Mature*				
Ctl	$2.65\pm 0.80$	$187.64\pm 18.16$	$18.0\pm 0.8$	$0.985\pm 0.007$
3 weeks	$0.65\pm 0.15$	$58.36 \pm 6.43$	$16.2\pm 1.2$	$0.955\pm 0.018$
6 weeks	$1.12\pm 0.14$	$95.36 \pm 8.21$	$15.4\pm 0.5$	$0.980\pm 0.005$
*Aging*				
Ctl	$0.82\pm 0.17$	$103.30\pm 13.04$	$19.6\pm 2.0$	$0.964\pm 0.016$
3 weeks	$0.92\pm 0.13$	$78.52 \pm 6.82$	$21.3\pm 3.6$	$0.973\pm 0.014$
6 weeks	$0.53\pm 0.17$	$70.64 \pm 7.41$	$18.8\pm 1.0$	$0.882\pm 0.031$
*Aged*				
Ctl	$0.67\pm 0.14$	$99.70\pm 9.47$	$17.6\pm 1.0$	$0.921\pm 0.024$
3 weeks	$0.19\pm 0.07$	$36.49\pm 7.74$	$21.8\pm 3.5$	$0.874\pm 0.046$
6 weeks	$0.91\pm 0.24$	$74.94\pm 9.79$	$16.4\pm 1.0$	$0.963\pm 0.021$

$^{*\mathrm{a}}$Significance with age ($p <0.001$); $^{*\mathrm{i}}$significance with injury ($p <0.001$); $^{*\mathrm{ai}}$significant age–injury interaction ($p<0.001$)

### Model sensitivity analysis and parameter determinability

#### SHR model

**Fig. 3:**
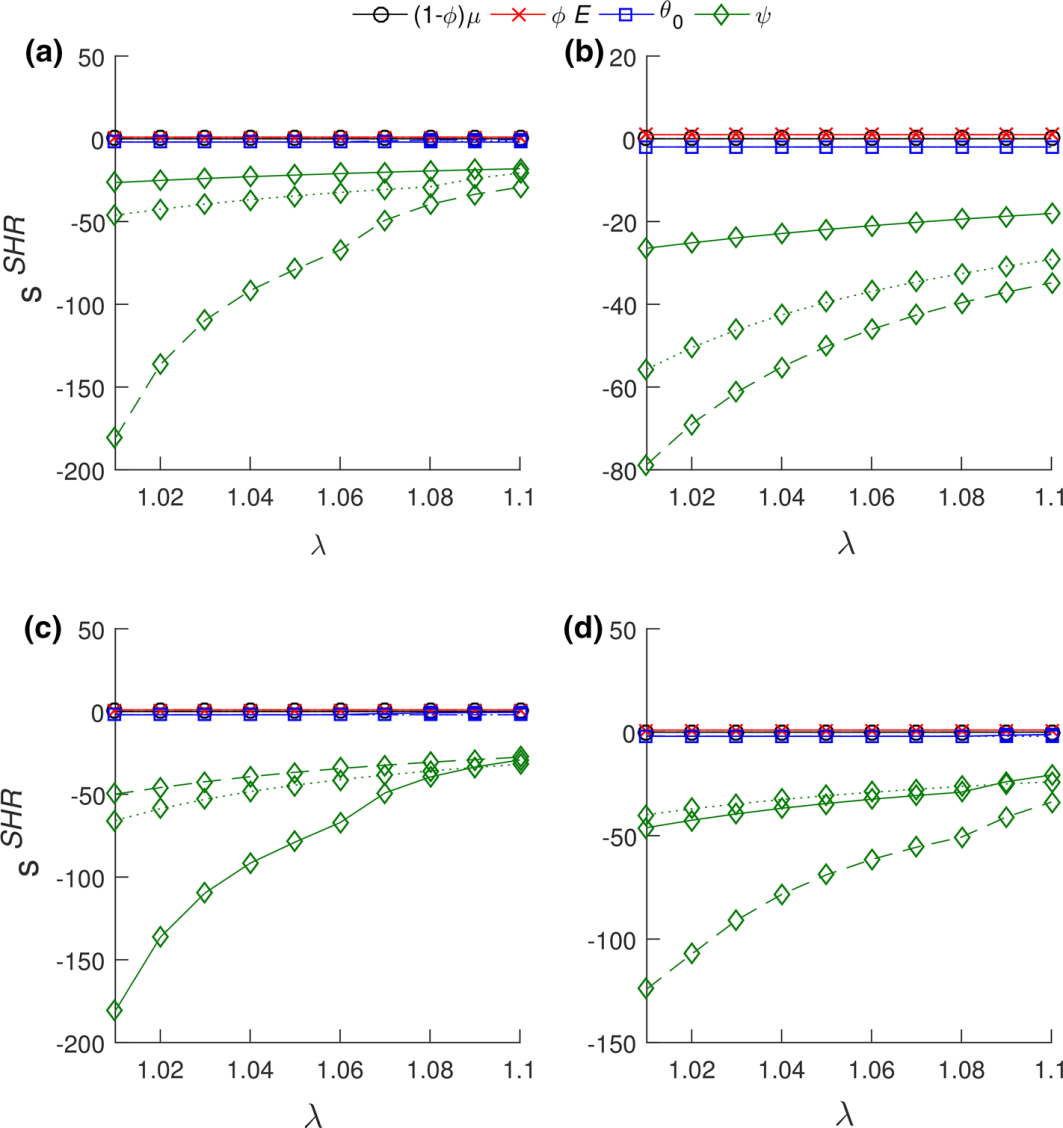
Sensitivity of the SHR model parameters in (**a**) aging—solid lines represent mature, dashed lines represent aging, and dotted lines represents aged **b** mature group healing, **c** aging group healing, and **d** aged group healing. In (**b**)–(**d**), solid lines represent uninjured, dashed lines represent 3 weeks post-injury and dotted lines represents 6 weeks post-injury. $\psi$, the fascicle alignment angle was the most influential

The fascicle alignment angle, $\psi$, was the most influential of the SHR model parameters across all ages and post-injury timepoints (Fig. 3). Dependence between the SHR model’s fibril Young’s modulus ($\phi E$) and crimp angle ($\theta _{o}$) was observed. The correlation between both parameters across all ages and healing timepoints was $\approx -\,1$.

#### GOH model

**Fig. 4:**
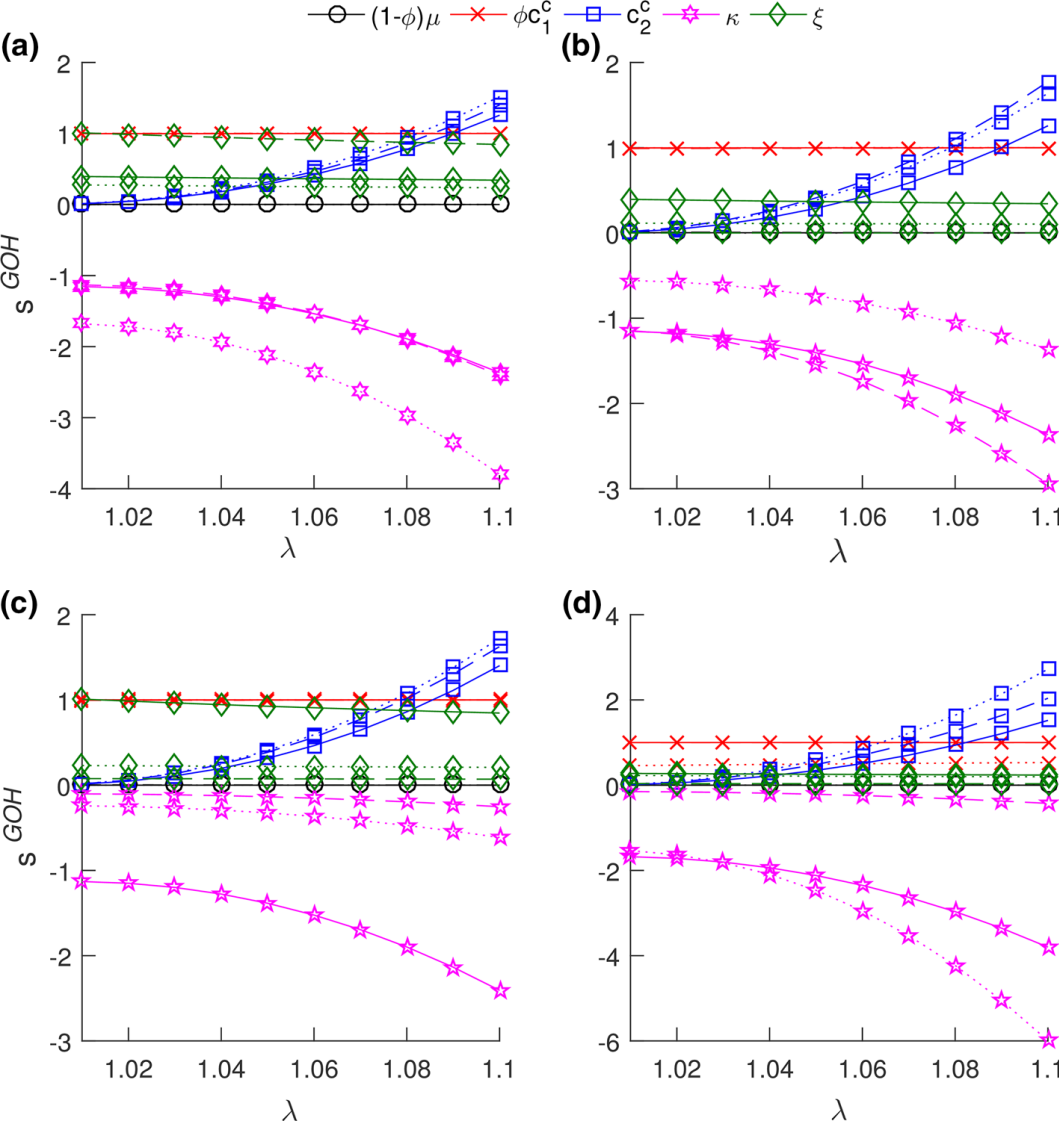
Sensitivity of the GOH model to its parameters during **a** aging—solid lines represent mature, dashed lines represent aging, and dotted lines represents aged groups, respectively, **b** mature group healing, **c** aging group healing, and **d** aged group healing. In (**b**)–(**d**), solid lines represent uninjured, dashed lines represent 3 weeks post-injury, and dotted lines represents 6 weeks post-injury. $\kappa$ was the most influential of the model parameters

$\kappa$ (measure of collagen fiber dispersion) of the GOH model was the most influential parameter to axial stress across all age groups and post-injury timepoints (Fig. 4). $\phi c_{1}^\mathrm{c}$ (modulus-like parameter) and $\xi$ (preferred collagen fiber orientation) exhibited dependence. The correlation coefficient of both parameters across all ages and post-injury timepoints was $\approx$ 1. The GOH model’s sensitivity to $c_{2}^\mathrm{c}$ exhibited an increase with stretch as a function of age and injury. ($1-\phi )\mu$ exhibited zero sensitivity as a function of age and injury.

**Fig. 5:**
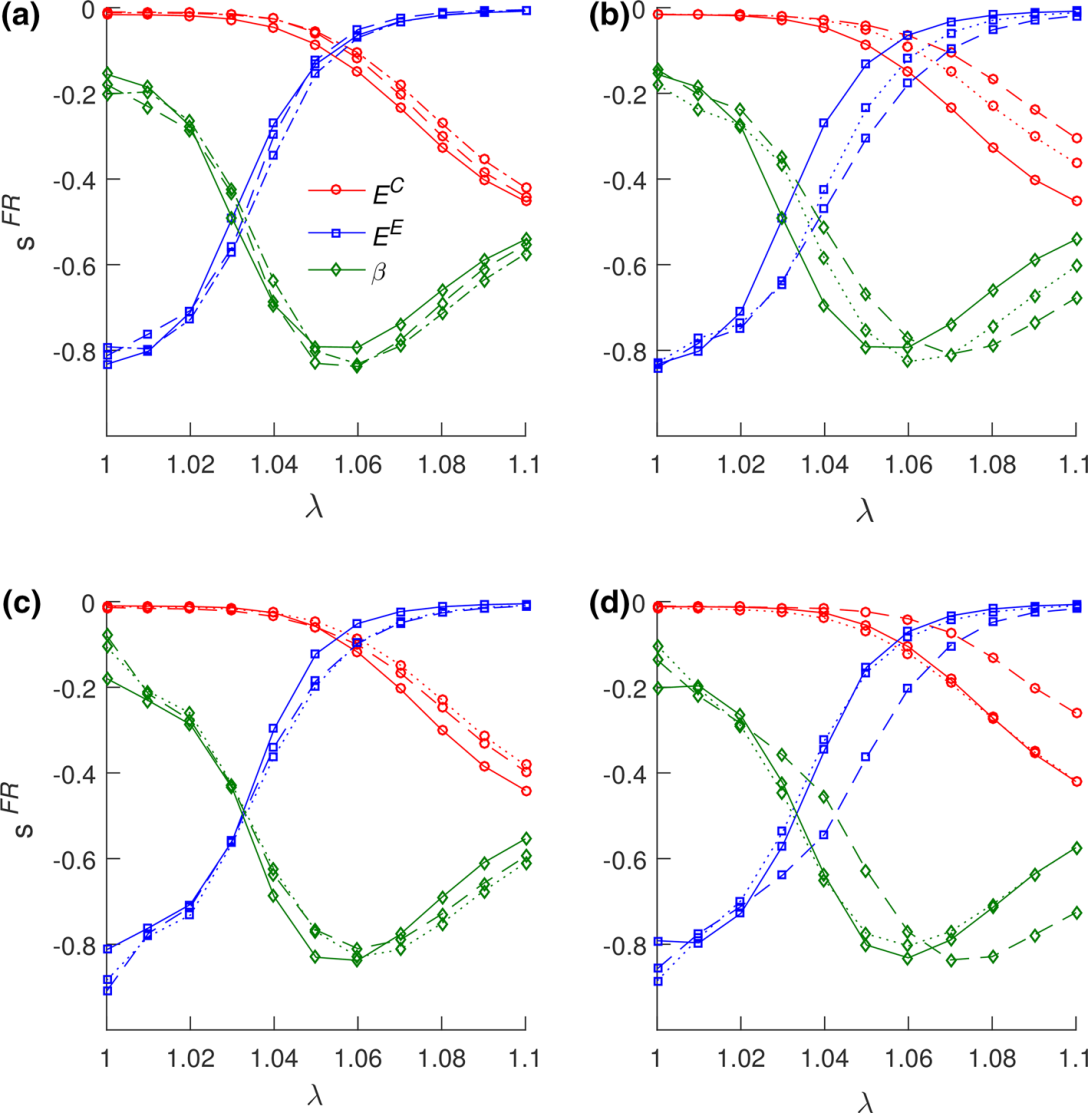
Sensitivity of the FR model in **a** aging—solid lines represent mature group, dashed lines represent aging, and dotted lines represents aged, **b** mature group healing, **c** aging group healing, and **d** aged group healing. In (**b**)–(**d**), solid lines represent uninjured, dashed lines represents 3 weeks post-injury, and dotted lines represents 6 weeks post-injury. The influence of the toe region modulus (blue) decreased with stretch as the tendon approached the estimated transition stretch. A switch in the influence of the moduli parameters is observable at the estimated transition stretch. The linear region moduli (red) became influential beyond the estimated transition stretch, though not reaching the same level of influence as that of the toe region modulus before the estimated transition stretch. A decrease in the influence of crimp (i.e., increase in the influence of $\beta$) is noticeable as the estimated stretch is approached. A delay in the estimated transition stretch due to injury is mostly observable in the mature group (**b**) and in the aged group (**d**)

#### FR model

The FR model parameters did not exhibit dependency and were all determinable (Fig. 5). A switch in the sensitivity of the FR model at the estimated transition stretch $\left( \lambda ^{*} \right)$ was observed (Fig. 5) between the toe region $(E^\mathrm{E})$ and linear region moduli $(E^\mathrm{C})$, with $E^\mathrm{E}$ more influential before $\lambda ^{*}$ and $E^\mathrm{C}$ beyond $\lambda ^{*}$. The influence of $\beta \,$ (crimp-related parameter) increased with stretch due to reduction in crimp up until $\lambda ^{*}$. Beyond $\lambda ^{*}$, the model sensitivity to $\beta$ decreased. A shift (delay) in $\lambda ^{*}$ during healing was observed, and this delay was largest between the uninjured and 3-week post-injury timepoints in the mature (Fig. 5b) and aged (Fig. 5d) groups.

### Statistical analysis

#### SHR model

**Fig. 6:**
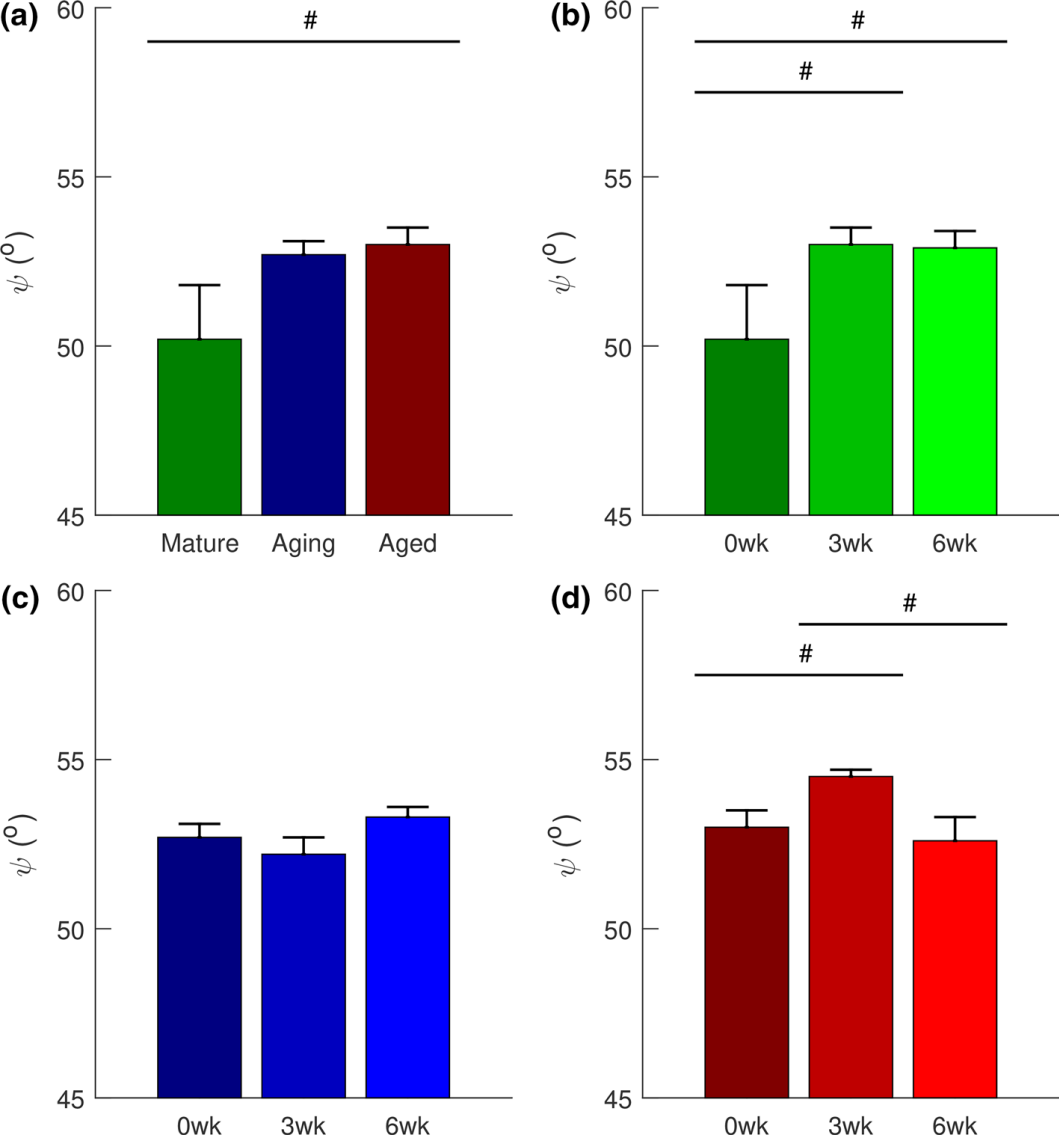
Results of post hoc tests on SHR model’s $\psi$ (fascicle alignment angle). **a** Effect of age on fascicle alignment of the uninjured subgroups. Age-dependent effects of injury on fascicle alignment within **b** the mature group, **c** the aging group, and **d** the aged group. $\psi$ exhibited trend toward decreased collagen fascicle alignment with age (**a**) and post-injury except in the aging group (**c**), which exhibited neither statistical significance nor trend with injury. Only the aged group exhibited improved fascicle alignment at 6 weeks. Data are presented as mean ± standard error. Statistically significant trend is denoted by # ($p<0.1$)

Fibril crimp angle, $\theta _{o}$ of the SHR model exhibited a trend toward statistical significance in the age–injury interaction ($p<0.1$) (Table 2). The fascicle alignment angle, $\psi$ exhibited statistical significance as a function of age ($p<0.05$), injury ($p<0.05$), and age–injury interaction ($p<0.05$) (Table 2). As a function of age, $\psi$ exhibited increasing trend toward significance ($p<0.1$) from mature to aged group (Fig. 6a). In the mature group, it exhibited an increasing trend toward significance ($p<0.1$) from 0 weeks to both 3 and 6 weeks timepoints (Fig. 6b). The aging group exhibited neither statistical significance nor a trend as a function of injury (Fig. 6c). $\psi$ exhibited an increasing statistical trend ($p<0.1$) from 0 to 3 weeks and a decreasing trend ($p<0.1$) from 3 to 6 weeks in the aged group (Fig. 6d).

**Fig. 7:**
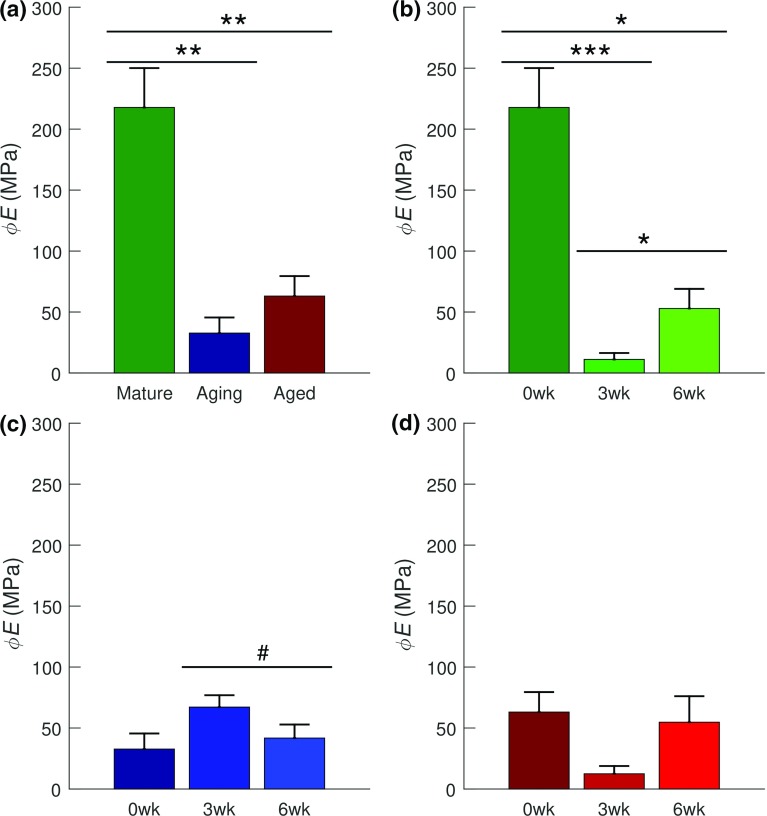
With revised constraints: $\theta _{0}\, \mathrm {\epsilon }\, \left[ 0^{\circ },{45}^{\circ } \right]$ and $\psi \, \mathrm {\epsilon }\, \left[ 0^{\circ },{10}^{\circ } \right]$, the results of post hoc tests on SHR model’s $\phi E$ (Fibril Young’s modulus). **a** Effect of age on the uninjured subgroups. Age-dependent effects of injury on fibril modulus within: **b** the mature group, **c** the aging group, and **d** the aged group. Fibril modulus reduced with age (**a**), and with injury except in the aging group (**c**), and in the aged group (**d**), which exhibited neither statistical significance nor trend with injury. Data are presented as mean ± standard error. Statistical significance is denoted by: *** ($p<0.001$), ** ($p<0.01$), and * ($p<0.05$) and significant trend is denoted by # ($p<0.1$)

**Fig. 8:**
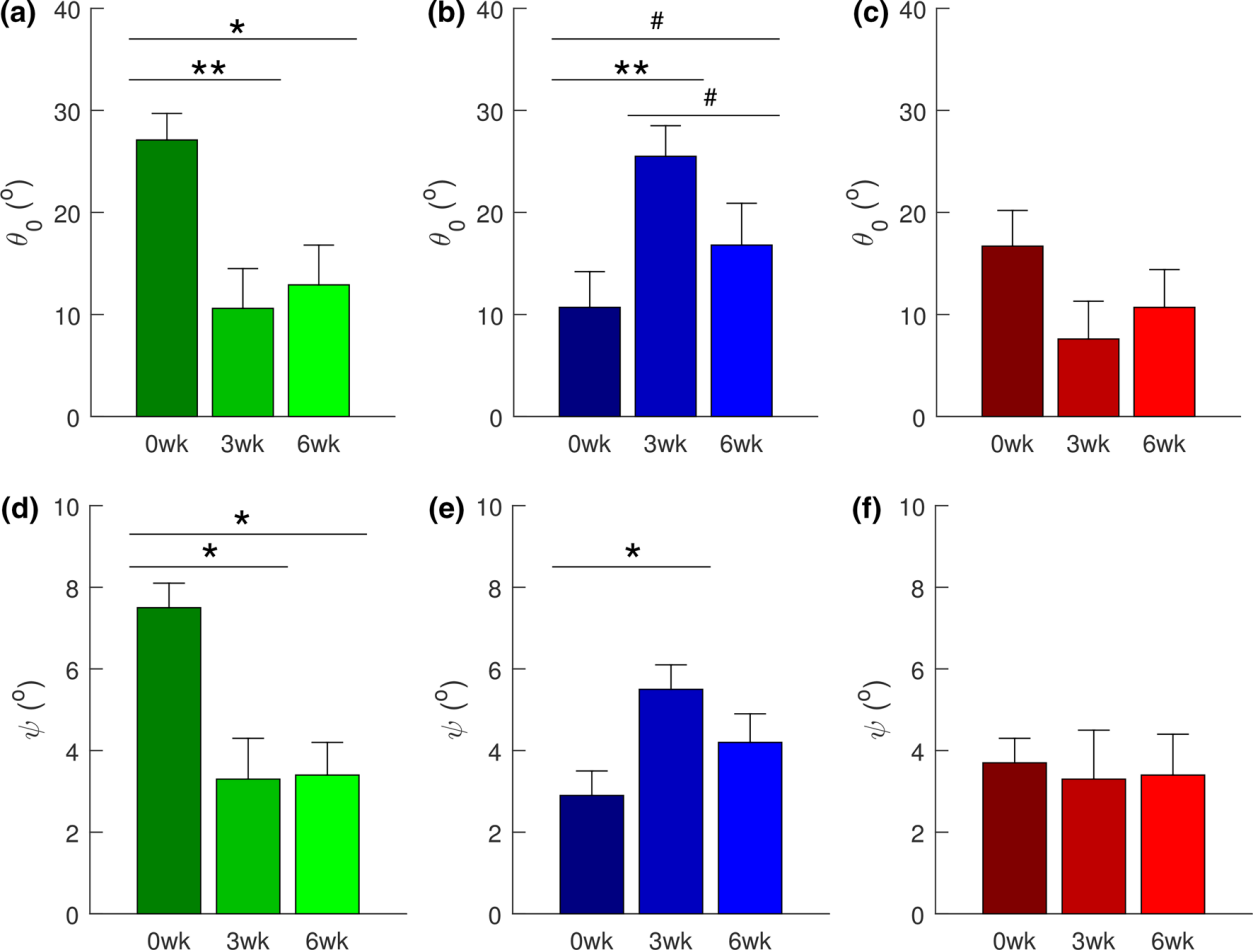
With revised constraints: $\theta _{0}\, \mathrm {\epsilon }\, \left[ 0^{\circ },{45}^{\circ } \right]$ and $\psi \mathrm {\, \epsilon }\, \left[ 0^{\circ },{10}^{\circ } \right]$, the results of post hoc tests on (top row) SHR model’s $\theta _{0}$ (Fibril crimp angle), and (bottom row) $\psi$ (fascicle alignment angle). Top row: age-dependent effects of injury within: **a** the mature group, **b** the aging group, and **c** the aged group. Crimp angle reduced post-injury (0–3 weeks) in the mature group (**a**) and increased post-injury in the aging group (**b**), which also exhibited trend toward decrease during healing (3–6 weeks). The aged group exhibited neither statistical significance nor trend post-injury. Bottom row: age-dependent effects of injury within: **d** the mature group, **e** the aging group, and **f** the aged group. Fascicle alignment reduced post-injury (0–3 and 6 weeks) in the mature group (**d**) and increased post-injury in the aging group (0–3 weeks) (**b**). The aged group exhibited neither statistical significance nor trend with injury (**f**). Data are presented as mean ± standard error. Statistical significance is denoted by: ** ($p<0.01$) and * ($p<0.05$) and significant trend is denoted by # ($p<0.1$)

With the revised constraints—$\theta _{0}\, \mathrm {\epsilon \, }\left[ 0^{\circ }, {45}^{\circ } \right]$ and $\psi \, \mathrm {\epsilon }\, [ 0^{\circ }, {10}^{\circ } ]$, the parameters: $\phi E$, $\theta _{o}$, and $\psi$ exhibited significant ($p<0.05$) age–injury interactions, while $\phi E$ also exhibited statistical significance as a function of age ($p<0.05$) and injury ($p<0.05$) (Table 3). With age, $\phi E$ decreased from the mature to the aging ($p<0.01$) and aged groups ($p<0.01$) (Fig. 7a). In the mature group, it decreased post-injury from 0 to 3 weeks ($p<0.001$) and 6 weeks ($p<0.05$) and it increased during healing from 3 to 6 weeks ($p<0.05$) (Fig. 7b). In the aging group, $\phi E$ exhibited a trend ($p<0.1$) toward decrease from 3 to 6 weeks (Fig. 7c). In the aged group, neither significance nor trend was exhibited (Fig. 7d). In the mature group, $\theta _{0}$ and $\psi$ decreased post-injury from 0 to 3 weeks ($\theta _{0}$: $p<0.01$ and $\psi$: $p<0.05$) and to 6 weeks (both: $p<0.05$) (Fig. 8a, d). In the aging group, both parameters increased post-injury from 0 to 3 weeks ($\theta _{0}$: $p<0.01$ and $\psi$: $p<0.05$), $\theta _{0}$ exhibited trend ($p<0.1$) toward increase from 0 to 6 weeks and toward decrease ($p<0.1$), during healing from 3 to 6 weeks (Fig. 8b, e). Neither $\theta _0$ nor $\psi$ exhibited significance or trend within the aged group (Fig. 8c, f).

#### GOH model

**Fig. 9:**
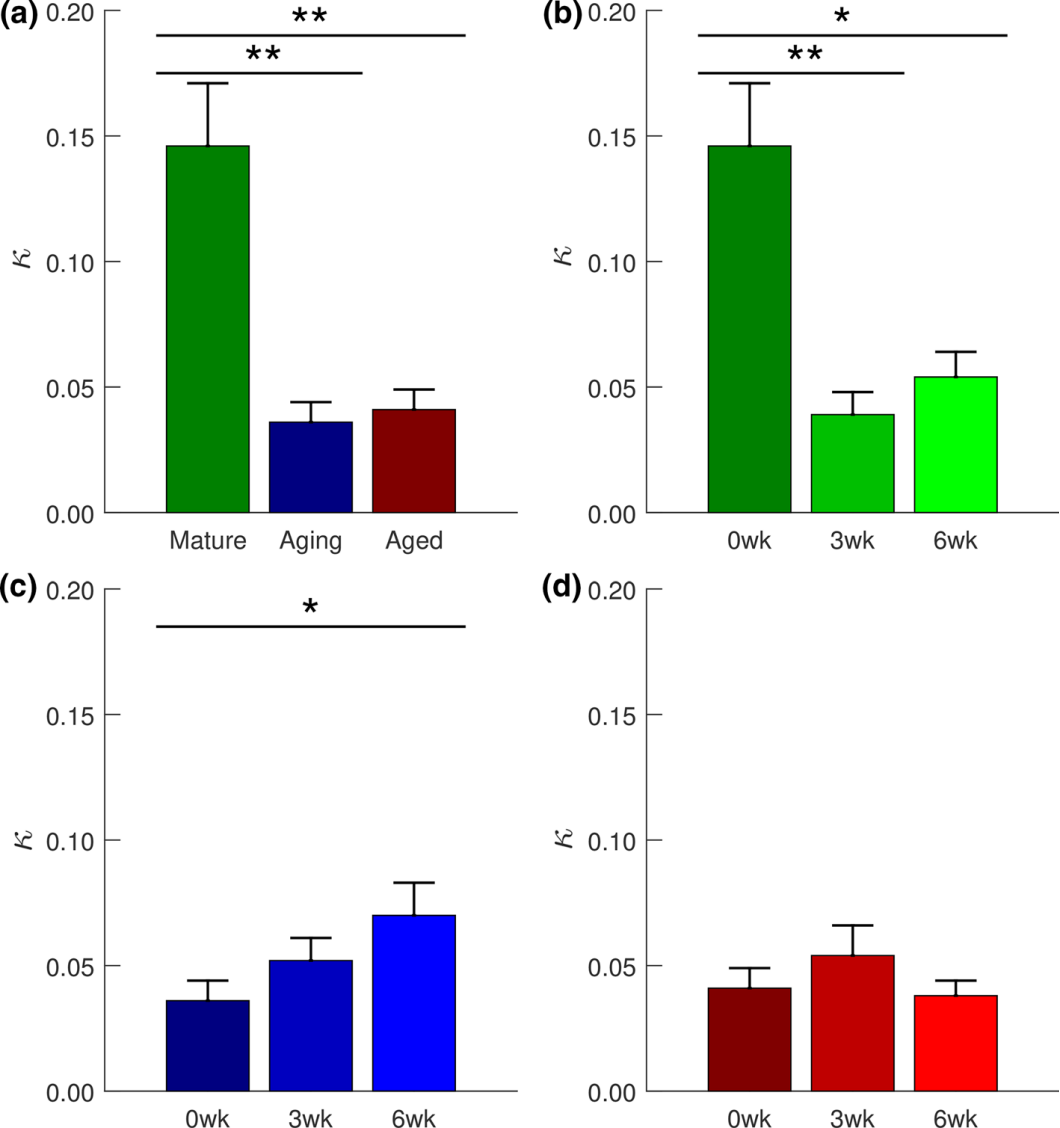
Results of post hoc tests on GOH model’s $\kappa$ (collagen dispersion). **a** Effect of age on collagen dispersion of the uninjured subgroups. Age-dependent effects of injury on collagen dispersion within: **b** the mature group, **c** the aging group, and **d** the aged group. Collagen dispersion reduced with age (**a**) and with injury except in the aging group (**c**) and in the aged group, which exhibited neither statistical significance nor trend with injury. Data are presented as mean ± standard error. Significance is denoted by: *** ($p<0.001$), ** ($p<0.01$), and * ($p<0.05$)

$\kappa$ of the GOH model exhibited statistical significance as a function of age ($p<0.01$), injury ($p<0.05$), and the interaction between both factors ($p<0.001$) (Table 4). A significant decrease in $\kappa$ (i.e., increased collagen fiber alignment) was observed with age: from mature to aging ($p<0.01$) and from mature to aged ($p<0.01$) (Fig. 9a). In the mature group, $\kappa$ decreased post-injury (0–3 weeks ($p<0.01$) and 0–6 weeks ($p<0.05$)) (Fig. 9b). In the aging group, however, $\kappa$ increased post-injury from 0 to 6 weeks ($p<0.05$) (Fig. 9c). No significant change in $\kappa$ was detected in the aged group (Fig. 9d).

**Fig. 10:**
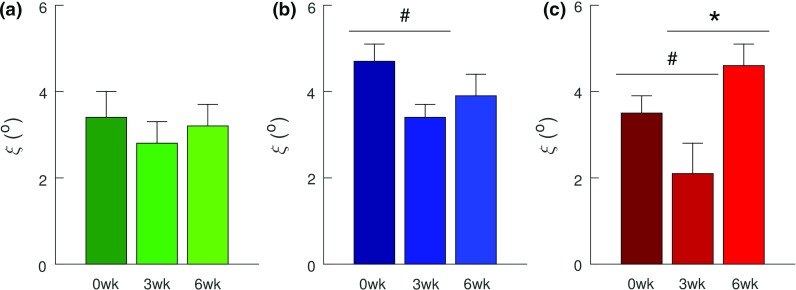
With revised constraint of $\xi \, \epsilon \, \left[ 0,{10}^{\circ } \right]$, results of post hoc tests on GOH model’s $\xi$ (preferred fiber direction). Effects of injury on preferred direction within: both aging (**b**) and aged (**c**) groups exhibited statistical trend toward decrease post-injury (0–3 weeks), the aged group (**c**) exhibited statistical significant increase during healing (3–6 weeks). The mature group exhibited neither statistical significance nor trend with injury. Data are presented as mean ± standard error. Significance is denoted by * ($p<0.05$); statistically significant trend is denoted by # ($p<0.1$)

With revised constraint for $\xi \, \epsilon \, \left[ 0^{\circ },{10}^{\circ } \right]$, $\xi$ exhibited statistical significance with injury ($p<0.05$) (Table 5); however, age and the age–injury interaction were not statistically significant. With injury in the aged group, $\xi$ increased ($p<0.05$) from 3 to 6 weeks (Fig. 10c). Statistical trends ($p<0.1$) indicated a decrease in $\xi \,$ in the aging (Fig. 10b) and aged groups (Fig. 10c) post-injury from 0 to 3 weeks.

#### FR model

**Fig. 11:**
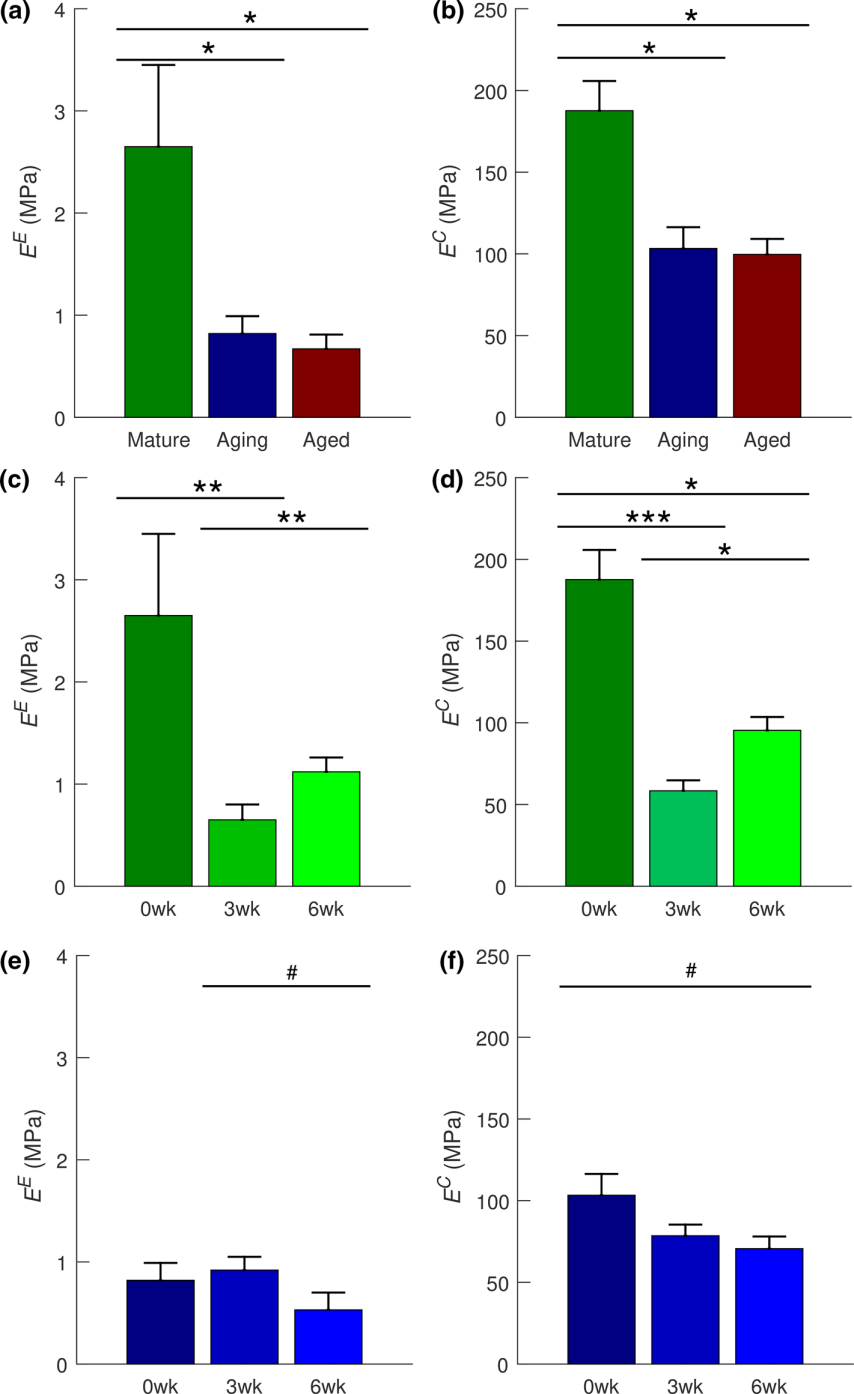
Results of post hoc tests on FR’s $E^\mathrm{E}$ and $E^\mathrm{C}$. **a**, **b** Effect of age on of the uninjured subgroups. Age-dependent effects of injury on FR moduli parameters within: **c**, **d** the mature group, **e**, **f** the aging group, and **g**, **h** the aged group. FR’s $E^\mathrm{E}$ and $E^\mathrm{C}$ decreased with age (**a**, **b**) and post-injury except $E^\mathrm{E}$ in the aging group (**c**). The aged group (**d**) may have exhibited seemingly the best healing capacity to 6 weeks post-injury when compared to the mature group (**b**). Data are presented as mean ± standard error. Significance is denoted by *** ($p<0.001$), ** ($p<0.01$), * ($p<0.05$), and trend by # ($p<0.1$)



FR model moduli parameters ($E^\mathrm{E}$ and $E^\mathrm{C}$) were statistically significant ($p<0.05$) in age, injury, and age–injury interaction (Table 6). With age, a statistically significant decrease in both moduli was recorded between the mature group and both the aging and aged groups ($p<0.05$ for both moduli and group pairs) (Fig. 11a, b). In the mature group, a significant decrease was observed in both moduli ($E^\mathrm{E}$: $p<0.01$, $E^\mathrm{C}$: $p<0.001$) post-injury (i.e., between 0 and 3 weeks timepoints). The decrease in both moduli was followed by a significant increase ($E^\mathrm{E}$: $p<0.001$, $E^\mathrm{C}$: $p<0.05$) during healing (i.e., 3–6 weeks post-injury). Within the mature group, $E^\mathrm{C}$ exhibited a statistically significance decrease ($p<0.05$) from 0 to 6 weeks timepoint, indicating non-recovery of pre-injury tensile properties (Fig. 11c, d). In the aging group, both moduli only exhibited a trend toward statistical significance ($p<0.1$). $E^\mathrm{E}$ decreased between 3 and 6 weeks ($p<0.1$) while $E^\mathrm{C}$ decreased between 0 and 6 weeks ($p<0.1$) (Fig. 11e, f). In the aged group, both moduli exhibited a significant decrease ($p<0.001$ for both) between 0 and 3 weeks and significant increase ($p<0.05$) from 3 to 6 weeks. $E^\mathrm{C}$ exhibited a trend toward a significant decrease ($p<0.1$) from 0 to 6 weeks, indicating non-recovery of pre-injury tensile properties (Fig. 11g, h).

## Discussion

### Model fits and age-dependent healing

Several studies on tendon aging have reported increase (Kubo et al. 2007; Nielsen et al. 1998; Shadwick 1990 Wood et al. 2011), decrease (Dressler et al. 2002; Karamanidis and Arampatzis 2006; Onambele et al. 2006; Stenroth et al. 2012), and no change (Couppé et al. 2009; Haut et al. 1992; Hubbard and Soutas-Little 1984) in tendon biomechanical properties. This lack of convergence on the impact of age on tendons can be attributed, among other factors, to the approach taken in computing mechanical properties. The use of microstructurally motivated models eliminates subjective investigator inputs, such as point at which a tangent is constructed for estimation of stiffness or elastic moduli from load–displacement or stress–strain curves, respectively (Couppé et al. 2009; Wood et al. 2011). Herein, all three candidate microstructurally motivated models reasonably described the mechanical response of murine patellar tendons with increasing age and during age-dependent healing (Fig. 1). Additionally, this work identified that the murine patellar tendon post-injury behavior of the aging group was different from that of the mature and aging groups (Fig. 11). This unique behavior was not observed in the published studies which investigated healing in these three age groups, although the studies did not employ microstructural model but rather statistically compared dynamic moduli computed at multiple frequencies (Dunkman et al. 2014a; Mienaltowski et al. 2016). Huegel et al. made similar observations in rat supraspinatus tendons treated with autologous cells (Huegel et al. 2016). Juvenile (4 weeks) and aged (16 months) rat supraspinatus tendons exhibited improved mechanical properties 8 weeks post-treatment, but no changes between treated and control limbs were observed in the adult (8 months) rats (Huegel et al. 2016). The lack of improvement in the adult group was hypothesized to be due to equilibrium between catabolic and metabolic processes (Huegel et al. 2016); this hypothesis may also be valid for the aging group in this study. The FR model moduli [toe moduli (Fig. 11c, g) and linear moduli (Fig. 11d, h)] showed statistically significant increase during healing in the mature and aged groups. However, the seeming recovery expressed by the aged group must be cautiously investigated as it may have resulted from accumulation of advanced glycation endproduct (AGE) cross-links within collagen fibrils (Holliday 1995; Murrell and Walton 2001). The accumulation of AGE cross-links may result in suboptimal remodeling with a stiffer tendon post-injury but of inferior mechanical integrity and more susceptible to injury (Bailey 2001; Reddy 2004). Future work is needed to quantify the evolving cross-links present in tendon during aging and age-dependent healing. The effects of collagen cross-links could be modeled within the GOH model via the addition of an effective orientation density function, which phenomenologically quantifies the space covered by the cross-links in-between collagen fibers (Sáez et al. 2014a, 2016). For the SHR model, the number of cross-links could be related to the fibril modulus. These methods, however, would introduce an additional parameter to be constrained, fitted, and validated.

### Parameter determinability

While the GOH model has been widely implemented in constitutive modeling of soft tissues, thus far, the inherent dependencies between its parameters have not been studied for describing patellar tendon mechanical response. The results of the parameter determinability test revealed dependent model parameter pairs for the GOH and SHR models. Dependent parameters are weakly determinable from the model due to strong correlation resulting from inherent relationship between such parameters. In such situations, additional experimental data or parameter constraints are needed to ensure strong determinability. For example, the dependence observed between $\phi E$ and $\theta _{o}$ in the SHR model may indicate the need for age-specific, refined (i.e., narrowed) bounds for the fibril elastic modulus, $\phi E$. Altered collagen fibril diameter distribution, specifically, a shift from a unimodal to bimodal distribution with age (Dunkman et al. 2013) and a positive post-injury skewness (Mienaltowski et al. 2016) have been observed in murine patellar tendons. Smaller diameter fibrils are thought to be newer fibrils that are yet to be laterally assembled into mature, larger-diameter fibrils (Birk et al. 1995; Dunkman et al. 2013, 2014a; Mienaltowski et al. 2016). The smaller fibrils are thought to be of inferior mechanical properties compared to the mature fibrils, which in addition contain non-enzymatic, trivalent cross-links such as pyrroles (Kuypers et al. 1992). These structural alterations with age and injury might indicate that the chosen upper bound for $\phi E$ should be lowered in line with the observed reduced steepness of the linear region of the experimental stress–stretch curve as a function of age and injury. Additional experimental data will be needed, however, to motivate such reduced upper bound.

Collagen dispersion has been shown to increase with age and post-injury (Dunkman et al. 2013, 2014a, b). As expected, $\psi$, the SHR model’s collagen fascicle alignment angle exhibited a statistical trend toward increase with age (Fig. 6a) and post-injury (Fig. 6b, d). However, surprisingly $\kappa$, the GOH model’s fiber dispersion parameter, exhibited a statistical decrease with age and post-injury in the mature group (Fig. 9a, b). These collagen dispersion results were compared to published histological findings from the same dataset the selected models were fitted to, circular standard deviation (Csd, unit: degrees), a different measure of collagen dispersion, was used to assess collagen fiber dispersion (Dunkman et al. 2013, 2014a, b). Csd exhibited statistically significant increase with age from the mature to the aged groups (Dunkman et al. 2013); in the mature group it exhibited statistical trend toward increase post-injury (Dunkman et al. 2014b). Csd, however, exhibited neither statistical significance nor trend in the aging group (Dunkman et al. 2014a) and was not measured in the aged group (Mienaltowski et al. 2016).

The unexpected decreases in collagen fiber dispersion $\kappa$, with age and post-injury, and its lack of agreement with histological data may have resulted from lack of physiologic bounds for the phenomenological parameters $\phi c_{1}^\mathrm{c}$ and $c_{2}^\mathrm{c}$ (Table 1). In a similar tendon healing study in rat Achilles tendons, which employed the GOH model, a different parameter-constraining approach was taken (Bajuri et al. 2016). The values of $\kappa$ were chosen for pre- and post-injury timepoints (days 3, 8, 14 and 21) with the goal of recovering hypothesized changes post-injury in the other GOH model parameters (Bajuri et al. 2016). Although the expected increase was observed in $\phi c_{1}^\mathrm{c}$ up until day 14, $c_{2}^\mathrm{c}$ unexpectedly decreased during healing. $\left( 1-\phi \right) \mu$ decreased as expected from day 3 to 8 and from day 14 to 21 but increased unexpectedly from day 8 to 14. Based on the study results, the approach taken in constraining $\kappa$ did not completely yield the expected trends (Bajuri et al. 2016). Hence, $\kappa$ should be quantified from experimental data when available (Gasser et al. 2006).

While the parameters of the FR model were determinable, the toe and linear region moduli exhibited significant changes with age and post-injury in an age-dependent manner (Fig. 11). These changes could have resulted from a combination of relative changes in both the amount and organization of structurally significant constituents—mostly fibrillar collagen type I. The FR model, however, does not offer any insight into either the amount or the organization of fibers. While this is a limitation, crimp angle of the collagen fibrils can be estimated through the parameter $\beta =1 /\varepsilon _\mathrm{max}^\mathrm{crimp}$, using either Eq. (2) (from the SHR model, with $\alpha =0)$ or equivalently, $\varepsilon _\mathrm{max}^\mathrm{crimp}=\left( \sec \theta _{0}\mathrm {-}1 \right)$ (Diamant et al. 1972). Hence, changes in crimp angle with age and post-injury could be determined through $\beta$, which, however, did not exhibit either statistical significance or trend in this study.

### Sensitivity analysis

Model sensitivity analysis indicated that the ground substance shear modulus, $\left( 1-\phi \right) \mu$, in the SHR and GOH models exhibited minimal influence on the tendon’s uniaxial tensile response (Figs. 3, 4). This may be attributed to the negligible direct contribution of the ground substance to tensile stress; however, it is also an inherent limitation in uniaxial tensile data. While tendons primarily transmit loads from muscle to bone along their long axis, many energy-storing tendons (e.g., the supraspinatus tendon) experience complex loading environments (Fang and Lake 2015; Lake et al. 2010; Luo et al. 1998; Nakajima et al. 2004). Hence, biaxial tensile or compression tests are needed to inform this parameter. Collagen volume fraction ($\phi$) for tendons is reported in literature for healthy intact tendons (Albright and Brand 1987), including the uninjured mature mouse (Dourte et al. 2013). Evolving collagen fraction with increasing age and during healing, however, is needed to physiologically constrain this parameter in the GOH and SHR models. Further, the inclusion of physiologically relevant bounds for $\phi$ may permit the delineation of compositional alterations and changes in material properties (Young’s modulus) due to matrix turnover, reorganization, or both. Additionally, estimating the collagen fiber dispersion parameter, $\kappa$, from histology or tensile uniaxial data (Hurschler et al. 1997) through the concentration parameter of the von Mises distribution (Gasser et al. 2006) may improve the model results. Thus, the inherent potential of microstructural models cannot be fully harnessed until relevant microstructural and histological data are available to inform physiologically relevant bounds for model parameters. Future work is needed to inform these microstructural parameters and improve the models’ physiologic relevance.

A parametric study of $\kappa$ through finite element simulations of uniaxial extension tests by Gasser et al. (2006) showed that $\kappa$ controlled the location of the transition point. A shift to the right (delay) in the transition point was observed on the load–displacement curves for human iliac artery adventitial strips as the value of $\kappa$ reduced. The results herein show a similar delay in transition stretch of the stress–stretch curves (Fig. 1) corresponding to observed decrease in $\kappa$ values as functions of age and injury (Fig. 9a, b). The relative changes in the model’s sensitivity to $\kappa$ as a function of age and injury are due to the changes in the estimated transition stretch. The results of the parametric study herein indicate that the sensitivity of the model to $\kappa$ increased negatively with stretch due to more fiber recruitment with increasing stretch. As fibers are re-aligned toward the direction of load and recruited, $\kappa$ decreases, resulting in increased longitudinal modulus and axial stress with stretch. The downward shift in $\kappa$ for the aged group may indicate a delay in the transition stretch (with a lesser $\kappa$). This is substantiated by the decrease in $\kappa$, indicating increased alignment and increased influence on the axial stress. While shifts (magnitude changes) in the sensitivity plot shapes were observed for the SHR and GOH model parameters due to aging and/or injury, the overall shapes (relative influence) generally remained constant (Figs. 3, 4).

### Changes with revised constraints for SHR and GOH structural parameters

Although the revision (narrowing) of the bounds of SHR structural parameters ($\theta _{o}$, $\psi$) resulted in a reduced model-to-data fit, the optimized fibril Young’s modulus values decreased to $<\,1$ GPa (Table 3), which are within the bounds commonly reported for collagen fibrils (Buehler 2008). Variability in modulus values may be attributed to various factors including species, tendon, fibril maturity, cross-links, hydration state, or experimental methods (Buehler 2008; Eppell et al. 2006). In view of the highlighted issues, the physiologically relevant range of fibril Young’s modulus is hard to determine. In addition, the fibril modulus would be expected to be some order of magnitude above that of the tendon (Sasaki and Odajima 1996); however, the optimized $\phi E$ values (Fig. 7) were similar to those of the linear region modulus (tendon-level), $E^\mathrm{C}$ obtained in the FR model (Fig. 11). Interestingly, however, the trends in their behavior as a function of age and in age-dependent healing are similar. This observed similarity further highlights the unique post-injury behavior exhibited by the aging group (Figs. 7c, 11e, f).

The narrowed bounds on the SHR structural parameters ($\theta _{o}$, $\psi$) also resulted in significant age-dependent behavior. Both parameters reduced post-injury in the mature group but increased post-injury in the aging group (Fig. 8). While increase in crimp angle post-injury in the aging group may be due to the presence of newly synthesized fibers with a relatively short strain cycle history (Legerlotz et al. 2014), collagen fiber dispersion and fascicle alignment angle are expected to increase post-injury. Hence, the behavior of $\theta _{o}$ and $\psi$ in the mature group was surprising (Fig. 8). Both parameters, however, did not exhibit statistical significance or trend with injury in the aged group.

Revision of the bounds for the preferred fiber direction, $\xi$ resulted in statistically significant changes in $\xi$, as opposed to the prior observed changes in $\kappa$. Further, significant changes in $\xi$ were present with respect to injury only, as opposed to those previously observed with age, injury, and the age–injury interaction (Tables 4, 5). The changes identified in $\xi$ with injury (Fig. 10), however, were unexpected as increase in preferred fiber direction angle has been observed post-injury (Riggin et al. 2014). Further investigation into the interaction between preferred fiber direction and collagen dispersion is needed as there may be a compensatory mechanism between these two parameters.

### G&R Implementation

The three models considered in this work have varied level of microstructural details. The SHR model provides insights at both the fibrillar and fascicular structural levels through parameters such as fibril modulus, crimp angle, and fascicle alignment angle. As alluded to in previous sections, there is however a gross lack of physiologic data for these parameters to motivate relevant bounds during model fitting and validation. In addition, constitutive relations that guide the changes in parameters during processes such as aging and healing need to be developed and validated. For these reasons, while the SHR model offers the most microstructural details, its implementation in a G&R framework at this time is premature. Although the FR model is the simplest and its parameters determinable, it was developed through implicit elasticity theory, i.e., $W\, =\, \hat{W}\, (stress,\, strain)$, hence will require modification to the current established G&R frameworks, which are developed within hyperelasticity theory. Also, unlike the SHR and GOH models, the FR parameters while being microstructural do not allow for delineation of changes in collagen amount, reorganization, and the combined effects of both. Hence, the GOH is perhaps the best candidate model for an initial model given the compromise between microstructural and phenomenological parameters, as well as the opportunity to leverage its progenitor model’s (Holzapfel et al. 2000) wide implementation in G&R frameworks for related, but different soft tissue models (Miller et al. 2014, 2015; Valentin and Humphrey 2009a). The need remains however, for the quantification of $\kappa$—collagen fiber dispersion from experimental data (Gasser et al. 2006).

### General limitations

This study is not without limitations. First, we proposed that the murine patellar tendon under axial quasi-static extension can be considered, as first approximation, an incompressible material. This is, however, an assumption and a potential limitation within our model, as high Poisson’s ratio values have been reported in tendons (Lynch et al. 2003). Hence, future work is needed to measure changes in volume during mechanical testing as a function of age and injury to expand the proposed framework. It has been shown, however, the incompressibility assumption is reasonably valid for initial constitutive frameworks (Vergari et al. 2011). Second, MATLAB’s* lsqnonlin* function employs a local search method. Although finding global minima was attempted by setting multiple sets of random numbers as the initial guesses to ensure global minimum was found, robust global optimization algorithms that are better than local solvers can be employed (Rios and Sahinidis 2013). Also, injured murine tendons follow the typical wound healing course with overlapping phases: the inflammatory phase   hours to days (Fukasawa et al. 1987; Marsolais et al. 2001), the proliferative phase ~days to weeks (Garner et al. 1989; Gelberman et al. 1991; Oshiro et al. 2003), and the remodeling phase   weeks to months (Oshiro et al. 2003). While this study identified potential microstructural parameters of interest to tendon age-dependent healing, additional timepoints are necessary to better understand and predict tendon healing. In particular, this study identified that collagen microstructural parameters, such as collagen organization, were statistically significant in the GOH and SHR models. Hence, we submit that additional timepoints in the late remodeling phase are needed to better understand the dynamics of collagen organization post-injury in order to design potential interventions. Further, additional timepoints are needed in the early inflammatory phase and quantification of collagen cross-link formation is recommended.

## Conclusion

To the authors’ knowledge, this work is the first to evaluate the changes in tendon mechanical properties both as a function of age and injury in an age-dependent manner using microstructurally motivated models. The models evaluated exhibited adequate descriptive capability in age-dependent healing of the murine patellar tendon and the results identified a unique post-injury behavior exhibited by the aging group compared to the mature and aged groups in the murine patellar tendon not reported previously. Additionally, a decline in mechanical response was observed as a function of injury and age, and statistical analysis on the model parameters indicated collagen organization as a significant factor in aging and healing, coinciding with prior experimental observations (Dunkman et al. 2013, 2014a, b). This study was also the first to highlight inherent dependencies between parameters of widely used hyperelastic models for describing patellar tendon mechanical response. Hence, this work highlighted the critical need for additional microstructural data to inform and validate model outputs thereby increasing models’ physiologic relevance. Such data are necessary to validate a G&R model for tendon aging and healing (Humphrey and Rajagopal 2002; Lanir 2015; Rao et al. 2003; Thompson 2013).
